# Temperature‐Dependent Root Responses to Water Deficit Modulate Biological Nitrogen Fixation and Rhizosphere Dynamics in Soybeans

**DOI:** 10.1111/ppl.71029

**Published:** 2026-07-22

**Authors:** Camila Domingos Cabral, Gladys Angélica Apaza‐Castillo, Adriana Sturion Lorenzi, Pedro Henrique Pedron Mattiuzzi, Anaila Amaral de Alencar, Maria Carolina Quecine, Moacir Tuzzin de Moraes, Flávio Henrique Silveira Rabêlo, Paulo Mazzafera, Tiago Tezotto

**Affiliations:** ^1^ Department of Soil Science, “Luiz de Queiroz” College of Agriculture (Esalq) University of São Paulo (USP) Piracicaba São Paulo Brazil; ^2^ Center for Nuclear Energy in Agriculture (CENA) University of São Paulo (USP) Piracicaba São Paulo Brazil; ^3^ Department of Genetics, “Luiz de Queiroz” College of Agriculture (Esalq) University of São Paulo (USP) Piracicaba São Paulo Brazil; ^4^ Department of Biology, Institute of Biology State University of Campinas (UNICAMP) Campinas São Paulo Brazil; ^5^ Institute of Science and Technology Federal University of São Paulo (UNIFESP) São José dos Campos São Paulo Brazil

**Keywords:** climate change, drought, nodulation, rhizosphere microbiome, soil warming, symbiosis

## Abstract

Although soil warming and water scarcity are frequently associated with reductions in soybean productivity and biological nitrogen fixation (BNF), their combined effects remain poorly understood. This study evaluated how soil temperature and water regime influence nodulation, BNF efficiency, plant physiology and metabolism, soil enzymatic activity, and rhizosphere microbial communities in soybean plants grown at two soil temperatures (24°C and 36°C) under two water regimes: well‐watered (WW) and water deficit (WD). The WD treatment was the main limiting factor, reducing plant growth, nodule number, and biomass, ureide accumulation, and integrated BNF indices. Surprisingly, nodular efficiency and photosynthetic rate were higher under WD. Root‐zone warming under adequate water availability promoted greater plant and nodule biomass, higher ureide accumulation, and increased integrated BNF efficiency despite a reduction in nodule number. In addition, soil warming increased malondialdehyde and citrate concentrations in shoots as well as nodular concentrations of N, P, K, S, and B. Soil enzymatic activities and rhizosphere bacterial community structure varied among treatments, whereas fungal communities remained relatively stable. Overall, water deficit structured BNF limitation, while soil warming modulated metabolic responses. These results indicate that water availability and soil temperature jointly regulate biological nitrogen fixation and soil–plant–microbe interactions in soybeans, highlighting the importance of considering these factors together when developing management strategies under climate change scenarios.

## 1 Introduction

Global food security is increasingly threatened by climate change and projected population growth (Janni et al. 2023; UN 2024). Due to the strong dependence of agriculture on temperature and precipitation patterns (Zhang et al. 2017), extreme events such as heatwaves and prolonged droughts have intensified constraints on productivity and agroecosystem stability (Sano and Oki 2022; Terán et al. 2024). In this context, soybean ( *Glycine max* (L.) Merrill) is strategically important because of its high protein content and economic relevance (Huang et al. 2023). However, its productivity is highly sensitive to climate variability, particularly elevated temperatures and water deficit (Janni et al. 2023; Qiao et al. 2023).

Under tropical conditions, biological nitrogen fixation (BNF) represents the main source of nitrogen sustaining soybean production (Ciampitti et al. 2021; Hungria and Nogueira 2023). Soybeans are considered one of the crops with the greatest contribution to BNF among grain‐producing species, with fixation rates reaching up to 450 kg N ha^−1^ (Hungria and Mendes 2015). Globally, soybeans contribute approximately 16.4 million tons of fixed N annually, accounting for nearly 77% of legume‐derived nitrogen in agricultural systems (Herridge et al. 2008), and field evidence indicates that more than 70% of crop N demand may be supplied through BNF (Maluk et al. 2023). In Brazil, replacing mineral nitrogen fertilizers with BNF in soybean production has generated estimated savings of US$15.2 billion and avoided emissions of approximately 183 million Mg CO_2_‐equivalent, highlighting the economic and environmental importance of this process for sustainable agriculture (Telles et al. 2023).

In legumes, BNF is mediated by rhizobia, predominantly members of the α‐proteobacteria (*Rhizobium, Bradyrhizobium, Mesorhizobium, Ensifer*—formerly *Sinorhizobium*, and *Azorhizobium*) and, less frequently, β‐proteobacteria (*Burkholderia* –often *Paraburkholderia*, *Cupriavidus*, and *Trinickia*; Rahimlou et al. 2021). Following root infection and symbiosome formation within nodules, a mutualistic association is established in which the plant provides C substrates, while the bacteria reduce atmospheric N_2_ to ammonia (NH_3_) via nitrogenase enzymes (Kazmierczak et al. 2020; Yang et al. 2022). Fixed ammonium (NH_4_^+^) is assimilated through the GS/GOGAT pathway (Ullah and Ali 2025), and in soybean it is predominantly exported as ureides through the purine pathway (Thu et al. 2020; Nguyen et al. 2021; Thu and Tegeder 2025).

Exposure of plants to abiotic stresses such as drought and high temperatures triggers complex signaling networks and extensive physiological and metabolic reprogramming. These responses include adjustments in carbon (C) and nitrogen (N) metabolism, activation of antioxidant systems, and the modulation of respiratory and redox processes (Hasanuzzaman et al. 2013; Tiwari et al. 2017; Seth and Sebastian 2024). Such physiological adjustments can directly influence symbiotic nitrogen fixation and nodule functioning. The efficiency of BNF is highly sensitive to soil physicochemical conditions (Zhang et al. 2024). Nitrogenase activity requires tightly regulated microaerobic conditions maintained by leghemoglobin and substantial energy input (Larrainzar et al. 2020; Harris et al. 2024). Both water deficit and elevated soil temperatures impair nodulation, rhizobial survival, and respiratory dynamics, constraining nitrogenase performance (Kuzma and Layzell 1994; Hungria and Vargas 2000; Pinto et al. 2023; da Silva et al. 2024; Yeremko et al. 2025; Li et al. 2025). Moreover, soil temperature and moisture regulate physicochemical and microbial processes that directly influence nutrient cycling and BNF (Novák and Hlaváčiková 2019; Li et al. 2022; Zhao et al. 2023).

Despite extensive research on shoot responses to stress, comparatively limited attention has been directed toward root‐level processes (Cohen et al. 2020, 2021). Roots act as primary sensors of thermal and hydric fluctuations and function as integrative hubs that coordinate systemic, hydraulic, hormonal, and redox signaling (Kalra et al. 2024; Jin et al. 2024; Gul et al. 2026). Although the interaction between heat and drought can aggravate plant damage (Zandalinas et al. 2018; Poudel et al. 2024), plant responses to multiple stresses are often non‐additive and may reflect specific adaptive mechanisms (Das et al. 2016).

However, integrative studies at the soil–root interface addressing BNF, plant physiology, metabolism, and soil microbiota remain scarce. Therefore, in this study we evaluated the effects of soil temperatures and water regimes on BNF, testing the hypothesis that these factors interact nonlinearly, with water availability primarily structuring symbiotic performance and soil warming modulating associated physiological, metabolic, nutritional, and edaphic responses. Water deficit structured BNF limitation, while soil warming modulated metabolic responses.

## 2 Material and Methods

### 2.1 Experimental Site and Environmental Conditions

The experiment was conducted in a greenhouse at the Luiz de Queiroz College of Agriculture (ESALQ/University of São Paulo), Piracicaba, SP, Brazil (22°42′30″ S, 47°38′00″ W), from October 15 to December 3, 2024. Environmental conditions followed the natural seasonal variation typical of the humid subtropical climate (Cwa), without artificial control of air temperature or humidity.

### 2.2 Soil Sampling and Characterization

Surface soil (0–20 cm) was collected from a pasture area in Piracicaba, SP (22°42′22″ S, 47°37′01″ W), and classified as an Oxisol with sandy loam texture. After air‐drying and passing through a 2 mm sieve, the soil was analyzed for pH, organic matter content, macro‐ and micronutrients, and particle‐size distribution (Table S1).

Based on the analytical results, soil acidity was corrected with calcium and magnesium carbonates to increase base saturation to 80%. The soil was moistened and incubated for 20 days. Subsequently, phosphorus (P), potassium (K), boron (B), copper (Cu), zinc (Zn), and manganese (Mn) were applied as a nutrient solution with concentrations of 100, 200, 1, 5, 5, and 1.5 mg dm^−3^, respectively. Macronutrients were supplied via KH_2_PO_4_ and KCl, whereas micronutrients were provided through H_3_BO_3_, CuSO_4_·5H_2_O, ZnSO_4_·7H_2_O, and MnSO_4_·H_2_O.

### 2.3 Experimental Design and Treatments

The experiment followed a 2 × 2 factorial design with 12 replicates, consisting of two soil temperatures (24°C and 36°C) and two water regimes: well‐watered (WW), with soil moisture maintained at approximately 80% of field capacity, and water deficit (WD), imposed by controlled irrigation restriction. Thus, the factorial arrangement used in this study included individual stress conditions (24°C + WD and 36°C + WW), the combined stress condition (36°C + WD), and the reference control treatment (24°C + WW), allowing direct comparisons among isolated and interacting effects of soil warming and water deficit.

### 2.4 Soil Temperature Control and Water Availability Treatments

Soil temperature was controlled using a heating system consisting of insulated polyethylene boxes connected to ultrathermostatic water baths, maintaining temperatures at 24°C or 36°C. Each box contained 5.3 L PVC cylinders (15 cm diameter × 30 cm height) filled with soil. Continuous circulation of heated water between the water baths and insulated boxes ensured stable thermal conditions around the pots.

Soil volumetric water concentration and temperature were continuously monitored using WET150 multiparameter sensors (Delta‐T Devices) connected to a GP2 datalogger programmed to record data at 5‐min intervals.

Sensors were calibrated under laboratory conditions using soil samples at different water contents and validated by the gravimetric method to establish soil‐specific calibration curves. During the experiment, sensors were installed at 0–10 and 20–30 cm depths (four replicates per treatment) to monitor soil water status and ensure maintenance of WW (~80% field capacity) or controlled WD. Prior to treatment initiation, soil was homogenized and adjusted to the target moisture content.

Soil temperature and water deficit treatments were initiated 9 days after emergence, at the V2 developmental stage. This approach was adopted to ensure uniform seedling establishment and experimental standardization before treatment application, since all plants were initially maintained under well‐watered conditions at 24°C (control) prior to the imposition of the 36°C and water deficit treatments.

In the WD treatment, plants received 70% of the total irrigation depth applied to WW plants, representing a 30% reduction in water supply. Water replenishment was guided by soil moisture readings from sensors installed at both depths, ensuring consistent maintenance of WW conditions and controlled imposition of WD throughout the experimental period. Plants were maintained under these conditions until the R3 reproductive stage.

### 2.5 Seed Sowing and Rhizobial Inoculation

The soybean cultivar NEO 531 I2X was sown at a depth of 3 cm, with three seeds per pot. Seeds were inoculated in the sowing furrow with *Bradyrhizobium japonicum* (strains SEMIA 5079 and 5080). The liquid inoculant used in this study had a concentration of 7.0 × 10^9^ CFU ml^−1^, with a recommended standard dose of 300 mL ha^−1^, diluted in water. Seedling emergence occurred 5 days after sowing, and thinning was performed 2 days later to keep one plant per pot.

### 2.6 Plant Sampling and Measurements

Plants were harvested 50 days after sowing (DAS), corresponding to the R3 stage. Shoots were collected at the cotyledonary node (5 cm above the soil surface), frozen in liquid nitrogen, stored at −80°C, freeze‐dried, weighed for dry mass determination, and ground for shoot nutritional analysis, ureide determination, malondialdehyde quantification, and metabolomic analyses. Leaf discs from the middle third were used to determine relative water content (RWC).

Roots were washed, nodules were detached, counted, oven‐dried at 60°C to constant mass, weighed, ground, and used for nodular nutritional analysis. Rhizosphere soil samples were collected from soil adhered to the roots and used for soil enzyme activity and microbial metataxonomic analyses.

#### 2.6.1 Nodulation Efficiency and Integrated Biological Nitrogen Fixation Indices

Nodular efficiency was determined as the ratio of shoot N accumulation (mg N plant^−1^) to nodule dry mass (g plant^−1^) and expressed as mg N g^−1^ of nodules. Specific nodular efficiency was calculated by dividing shoot N accumulation by the total number of nodules, expressed as mg N nodule^−1^. The integrated BNF efficiency index (iBNF) was computed as the product of shoot N accumulation and ureide content in the shoots, normalized by nodule dry mass, and expressed in relative units (mg^2^ g^−1^).

### 2.7 Nutrient Analysis

#### 2.7.1 Macro and Micronutrients in Nodules

P, K, calcium (Ca), magnesium (Mg), sulfur (S), B, Cu, iron (Fe), Mn, Zn, molybdenum (Mo), nickel (Ni), silicon (Si), cobalt (Co), and aluminum (Al) were quantified after HNO_3_–H_2_O_2_ digestion in a closed microwave system following standard procedures for plant tissue analysis (Miller 1998). Elemental concentrations were determined by inductively coupled plasma optical emission spectrometry (ICP‐OES) as described by Jones (2001) and Hou et al. (2021).

#### 2.7.2 Total Nitrogen in Shoots and Nodules

Total N concentration in shoots and nodules was determined using the Kjeldahl method. Ground samples were digested with sulfuric acid in the presence of appropriate catalysts, followed by neutralization, distillation, and titration to quantify ammonium ions derived from organic nitrogen, according to the procedures described by Silva (2009) and AOAC (2005).

### 2.8 Physiological and Biochemical Analyses

Relative leaf water content (RWC) was determined according to Weatherley (1950) using fully expanded leaf discs. Fresh mass, turgid mass after 24 h rehydration, and dry mass after drying at 65°C were recorded. RWC was calculated as the ratio between water content at sampling and total tissue water‐holding capacity, expressed as a percentage.

Gas exchange parameters were measured on fully expanded leaves from the middle third of plants at the R2–R3 stages using a portable photosynthesis system (GFS‐3000, Heinz Walz GmbH) equipped with a standard leaf chamber (3010‐S) and LED light source (3040‐L). Measurements were conducted in the morning (08:00–11:00 h). The central leaflet of a healthy, fully expanded trifoliate leaf was clamped in the chamber and allowed to stabilize for approximately 7 min before data acquisition. Chamber conditions were maintained at a CO_2_ concentration of 400 μmol mol^−1^, air flow of 750 μmol s^−1^, photosynthetically active radiation (PAR) of 1200 μmol m^−2^ s^−1^, relative humidity of 65%, and temperature of 24°C.

Ureide (allantoin) concentrations in shoots were determined colorimetrically according to Goos et al. (2015) using the 2,3‐butanedione monoxime–thiosemicarbazide (BDM–TSC) reaction. Compounds were extracted from freeze‐dried and ground shoot tissue using hot water (0.2 g tissue in 10 mL ultrapure water at 90°C for 30 min). Extracts were first reacted with NaOH and subsequently with the BDM–TSC color reagent under heating, and absorbance was measured at 525 nm. Concentrations were calculated from an allantoin standard curve and expressed as mg ureide‐N kg^−1^ dry matter.

Shoot lipid peroxidation was assessed by determining malondialdehyde (MDA) content using the thiobarbituric acid (TBA) method described by Heath and Packer (1968), adapted for freeze‐dried tissues. Absorbance was measured at 532 nm, with correction at 600 nm, and MDA concentration was calculated using the molar extinction coefficient of the MDA–TBA complex. Results were expressed as nmol g^−1^ dry matter.

### 2.9 Soil Enzymatic Activities

The activities of β‐glucosidase, acid phosphatase, and arylsulfatase were determined in rhizospheric soil according to Tabatabai (1994), Tabatabai and Bremner (1969), and Spencer (1958), respectively, with minor adaptations. Soil samples (1 g) were incubated with p‐nitrophenyl‐derived substrates at 37°C for 1 h, and the released p‐nitrophenol was quantified spectrophotometrically at 420 nm for β‐glucosidase and 410 nm for acid phosphatase and arylsulfatase. Enzyme activities were expressed as μmol p‐nitrophenol g^−1^ h^−1^.

### 2.10 Targeted Metabolomic Analysis

Targeted metabolomic analyses were performed to quantify the metabolites proline, γ‐aminobutyric acid (GABA), aspartate, pyruvate, malate, citrate, succinate, valine, and adenosine using ultra‐high‐performance liquid chromatography coupled with tandem mass spectrometry (UHPLC–MS/MS) operating in a positive electrospray ionization mode with selected reaction monitoring (SRM). Chromatographic separation was achieved using an F5‐phase column under a gradient of water and acetonitrile acidified with 0.1% formic acid. Freeze‐dried plant material was extracted using an aqueous methanol–isopropanol solution containing an isotopically labeled internal standard. Extracts were ultrasonicated, centrifuged, and filtered prior to injection. Quantification was performed using weighted linear calibration curves (1/*X* ^2^).

### 2.11 Rhizosphere Metataxonomic and Bioinformatic Analysis

DNA from rhizospheric soil was extracted using the DNeasy PowerSoil Pro kit (QIAGEN). The bacterial community was amplified by a two‐step PCR targeting the V3–V4 regions of the 16S rRNA gene using the primer pair 341F and 806R (Zhang et al. 2023), and fungal communities were characterized by amplification of the ITS region using the primer pair ITS1 and ITS2. Libraries were prepared using the Rapid Plus DNA Library Preparation Kit and sequenced on the Illumina NovaSeq 6000 platform.

Raw sequences were processed using the DADA2 pipeline (Callahan et al. 2016) for denoising and generation of amplicon sequence variants (ASVs). Taxonomic assignment was performed using the SILVA v138.1 NR99 database for 16S sequences and the UNITE v10.0 database for ITS sequences. ASVs assigned to chloroplasts or mitochondria were removed. Downstream data organization, filtering, and phylogenetic analyses were conducted using the *phyloseq* package (R).

Bacterial (16S rRNA V3–V4) and fungal (ITS) amplicon sequencing datasets are publicly available in the European Nucleotide Archive (ENA) under BioProject accession numbers PRJEB108728 and PRJEB108779, respectively.

### 2.12 Statistical Analysis

Statistical analyses were performed using Jamovi software (version 2.7.17). Biometric, physiological, nutritional, enzymatic, and metabolomic data were analyzed using two‐way analysis of variance (ANOVA), considering soil temperature and water regime as fixed factors. When significant effects were detected, means were compared using Student's *t*‐test. Differences were considered significant at *p* ≤ 0.05.

Principal component analysis (PCA) was also performed to explore multivariate treatment patterns using centered and scaled data matrices. PCA results were visualized as biplots with normalized variables. In all analyses, a significance level of 5% (*p* ≤ 0.05) was adopted, and results are presented as mean ± standard error when appropriate. Correlation matrices were generated using Pearson's correlation coefficients and visualized as heatmaps.

In addition, metataxonomic analyses were conducted in the R environment (v4.3). Alpha diversity indices (Chao1 and Shannon) were evaluated using the Kruskal–Wallis test followed by Dunn's post hoc test with *p*‐value adjustment. Beta diversity based on Bray–Curtis distances was analyzed by PERMANOVA (function adonis, *vegan* package) and visualized using non‐metric multidimensional scaling (NMDS) in *phyloseq*. Mantel tests with 999 permutations were performed using the mantel function in vegan to evaluate the association between soil chemical attributes and dominant bacterial genera. Prior to testing, Pearson's correlations among variables were calculated with the Hmisc package (Harrell Jr 2025) to examine inter‐variable relationships.

## 3 Results

### 3.1 Growth, Biometric, and Nodulation Responses to Soil Temperature and Water Regime

**FIGURE 1:**
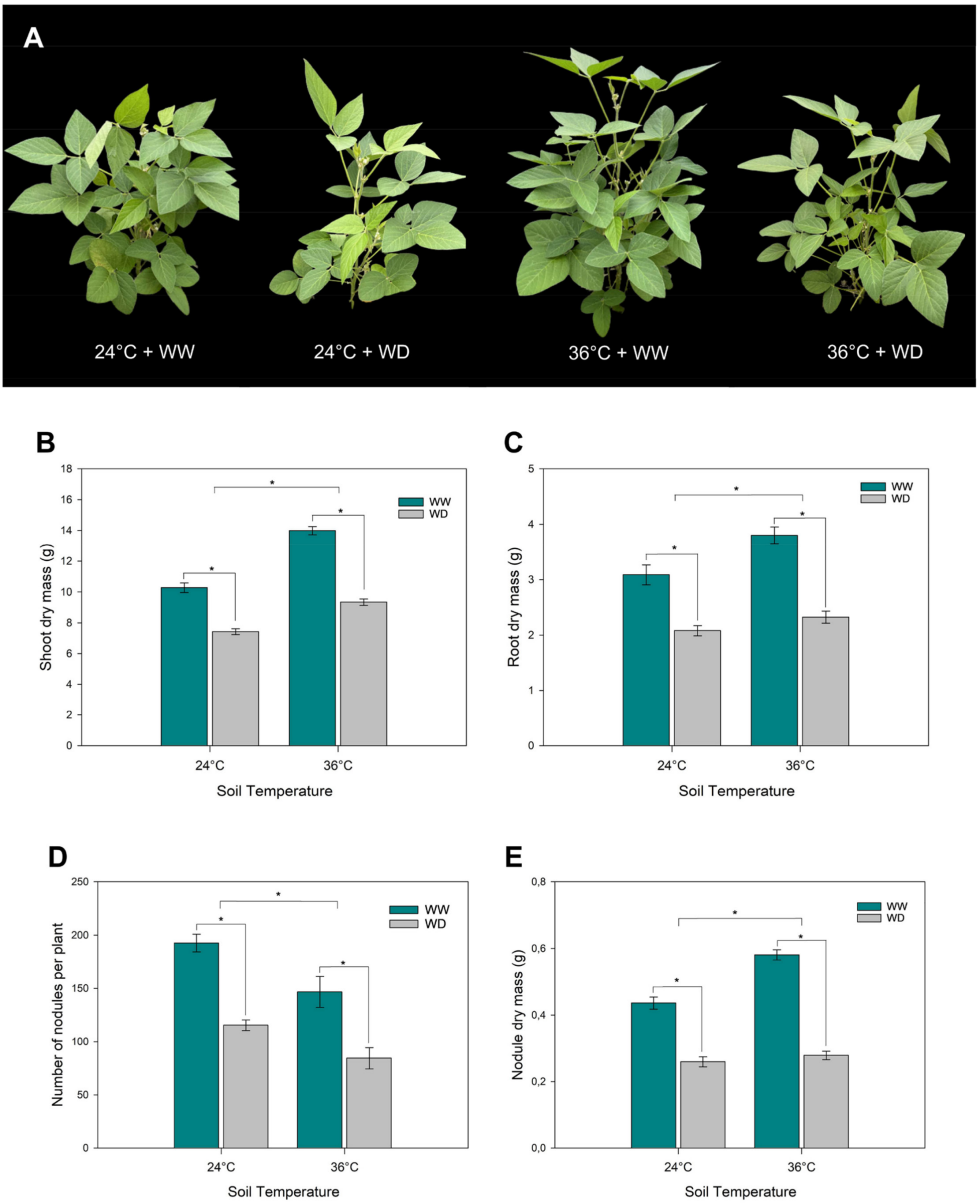
Effects of soil temperatures and water regimes on plant growth and nodulation. (A) Representative plants grown under four treatments: 24°C + well‐watered (WW), 24°C + water deficit (WD), 36°C + WW, and 36°C + WD. (B) Shoot dry mass, (C) root dry mass, (D) number of nodules per plant, and (E) nodule dry mass as affected by soil temperature (24°C and 36°C) and water regime (WW and WD). Bars represent mean ± standard error (*n* = 12). Asterisks indicate significant differences between treatments according to Student's *t*‐test (*p* ≤ 0.05).

The visual assessment of the plants revealed marked differences in growth among treatments (Figure 1A). Plants cultivated at 36°C under well‐watered conditions exhibited greater shoot development and canopy expansion compared with those grown at 24°C. In contrast, water restriction visibly reduced plant stature at both temperatures.

Shoot dry mass was significantly influenced by soil temperature and water availability (Figure 1B). Under well‐watered conditions, plants grown at 36°C accumulated greater biomass than those cultivated at 24°C. In contrast, water restriction significantly reduced shoot dry mass at both temperatures. Nevertheless, plants subjected to 36°C + WD maintained higher values than those observed under 24°C + WD, indicating that the stimulatory effect of elevated soil temperature on biomass accumulation persisted even under WD conditions.

Root dry mass followed a similar trend (Figure 1C), increasing at 36°C under well‐watered conditions, but declining significantly under water deficit at both temperatures. Nevertheless, plants grown at 36°C under water restriction maintained greater root biomass than those at 24°C, indicating enhanced root growth resilience under elevated temperature. Nodulation was markedly affected by the interaction between soil temperature and water availability (Figure 1D), with the highest nodule number observed at 24°C under well‐watered conditions. Elevated temperature reduced nodulation, and water deficit further intensified this decline, particularly under the combined 36°C + WD treatment. Nodule dry mass increased at 36°C under well‐watered conditions compared with 24°C, with elevated temperature stimulating nodule growth when water was adequate (Figure 1E). However, water deficit markedly reduced nodule biomass at both temperatures, with a more pronounced decline at 36°C, demonstrating the high sensitivity of nodules to water limitation.

Elevated soil temperature enhanced shoot, root, and nodule growth under adequate water supply, whereas water deficit considerably limited overall plant development. Under combined stresses, nodulation declined more sharply than vegetative growth at 36°C. PCA identified these traits as the biometric variables most closely associated with BNF performance. Consequently, shoot dry mass, root dry mass, nodule number, and nodule dry mass were defined as the primary indicators linking plant growth and BNF. Additional growth parameters exhibiting significant differences in the *t*‐test are provided in the Table S2.

### 3.2 Efficiency of Biological Nitrogen Fixation in Response to Soil Temperatures and Water Regimes

**FIGURE 2:**
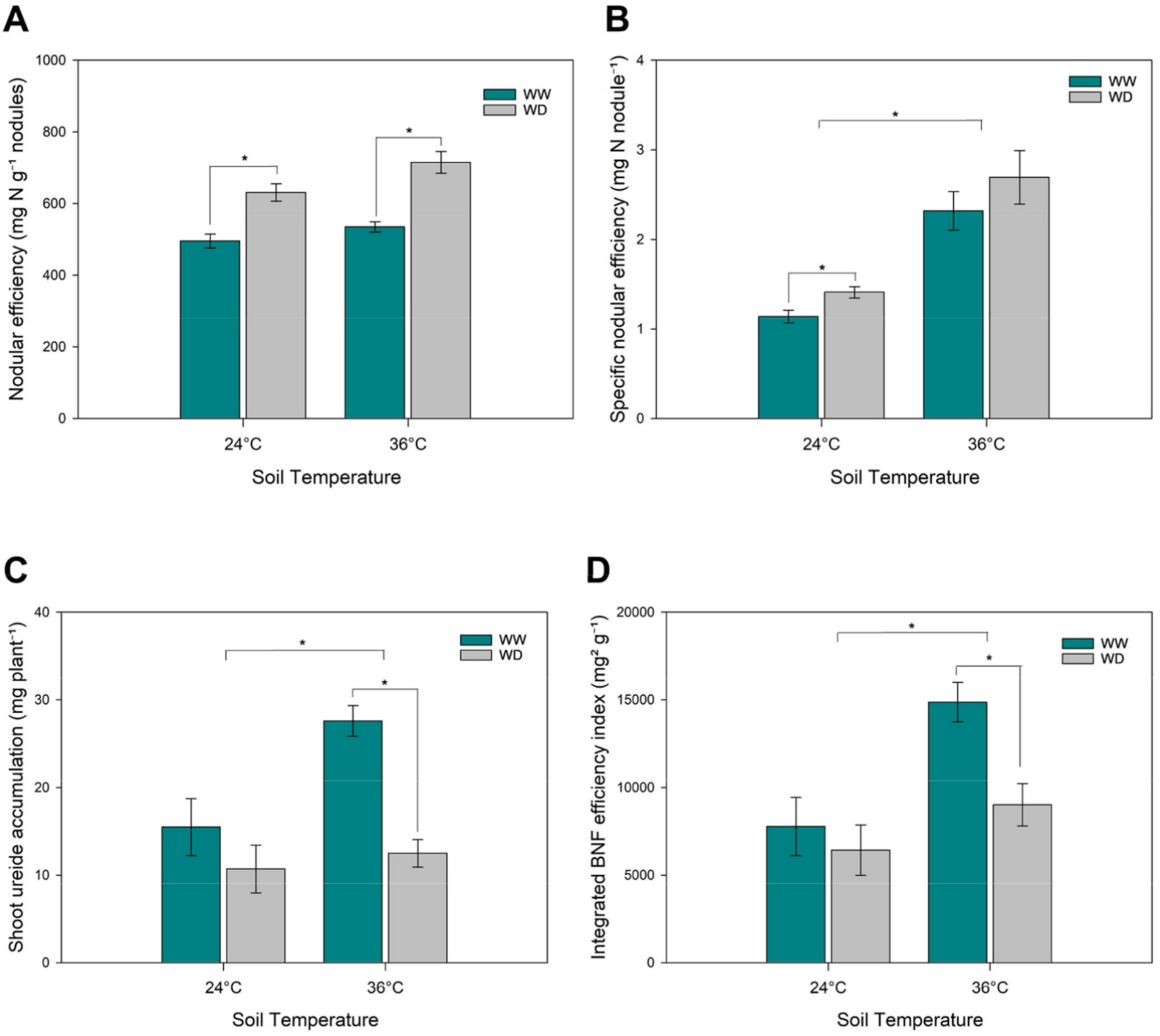
Effects of soil temperatures (24°C and 36°C) and water regimes (WW, well‐watered; WD, water deficit) on variables associated with biological nitrogen fixation (BNF) efficiency. (A) Nodule efficiency (mg N g^−1^ nodules). (B) Specific nodular efficiency (mg N nodule^−1^). (C) Shoot ureide accumulation (mg plant^−1^). (D) Integrated BNF efficiency index (mg^2^ g^−1^). Bars represent mean ± standard error (*n* = 12). Asterisks indicate significant differences between treatments according to Student's *t*‐test (*p* ≤ 0.05).

Variables associated with the efficiency of BNF exhibited contrasting responses to soil temperature and water availability (Figure 2). Nodule efficiency was significantly influenced by water regime, with higher values recorded under water deficit at both soil temperatures (Figure 2A), whereas soil temperature alone did not promote significant changes in this parameter.

Specific nodular efficiency was affected by both soil temperature and water regime in a temperature‐dependent manner (Figure 2B). Values at 36°C were significantly higher than those recorded at 24°C. The effect of water deficit was evident only at 24°C, where plants subjected to restricted irrigation exhibited greater N accumulation per nodule compared with well‐watered plants. In contrast, no significant differences between water regimes were detected at 36°C.

Ureide accumulation per plant, employed as an indicator of the flux of fixed N exported from nodules, was also significantly influenced by soil temperature and water regime (Figure 2C). Plants grown at 36°C exhibited greater total ureide accumulation than those cultivated at 24°C, indicating enhanced overall BNF activity under elevated temperature. Water deficit significantly reduced ureide accumulation, with a more pronounced decline at 36°C, consistent with the reductions observed in nodule number and biomass (Figure 1).

When analyzed in an integrated manner, the combined BNF efficiency index revealed marked differences among treatments (Figure 2D). The highest values were recorded in plants grown at 36°C under well‐watered conditions, whereas water restriction significantly decreased this index at the same temperature. In addition, plants subjected to 36°C combined with water deficit maintained higher values than those observed under 24°C with water deficit, showing that elevated soil temperature partially alleviated the adverse effects of water deficit on overall BNF efficiency.

The responses observed for BNF efficiency variables were consistent with shoot nitrogen concentration (Nsh) and shoot nitrogen accumulation (Nacc) presented in Figure S1. Although Nsh remained relatively stable across soil temperatures, Nacc was significantly higher in plants cultivated at 36°C, particularly under well‐watered conditions. This increase can be attributed to the enhanced shoot biomass accumulation observed under elevated temperature (Figure 1), reinforcing the close association between plant growth and BNF efficiency.

Ureide concentration in shoot dry matter and total ureide accumulation per plant (Figure S1) further supported the patterns observed in Figure 2. The variations detected in these parameters indicate that differences in BNF efficiency were associated not only with alterations in nodule activity but also with changes in the plant's capacity to assimilate, transport, and redistribute fixed N.

Furthermore, these findings demonstrate that soil temperature and water availability interactively modulate nodule efficiency and the contribution of individual nodules to BNF performance, in close association with shoot growth and nitrogen accumulation.

### 3.3 Gas Exchange and Oxidative Stress in Response to Soil Temperatures and Water Regimes

**FIGURE 3:**
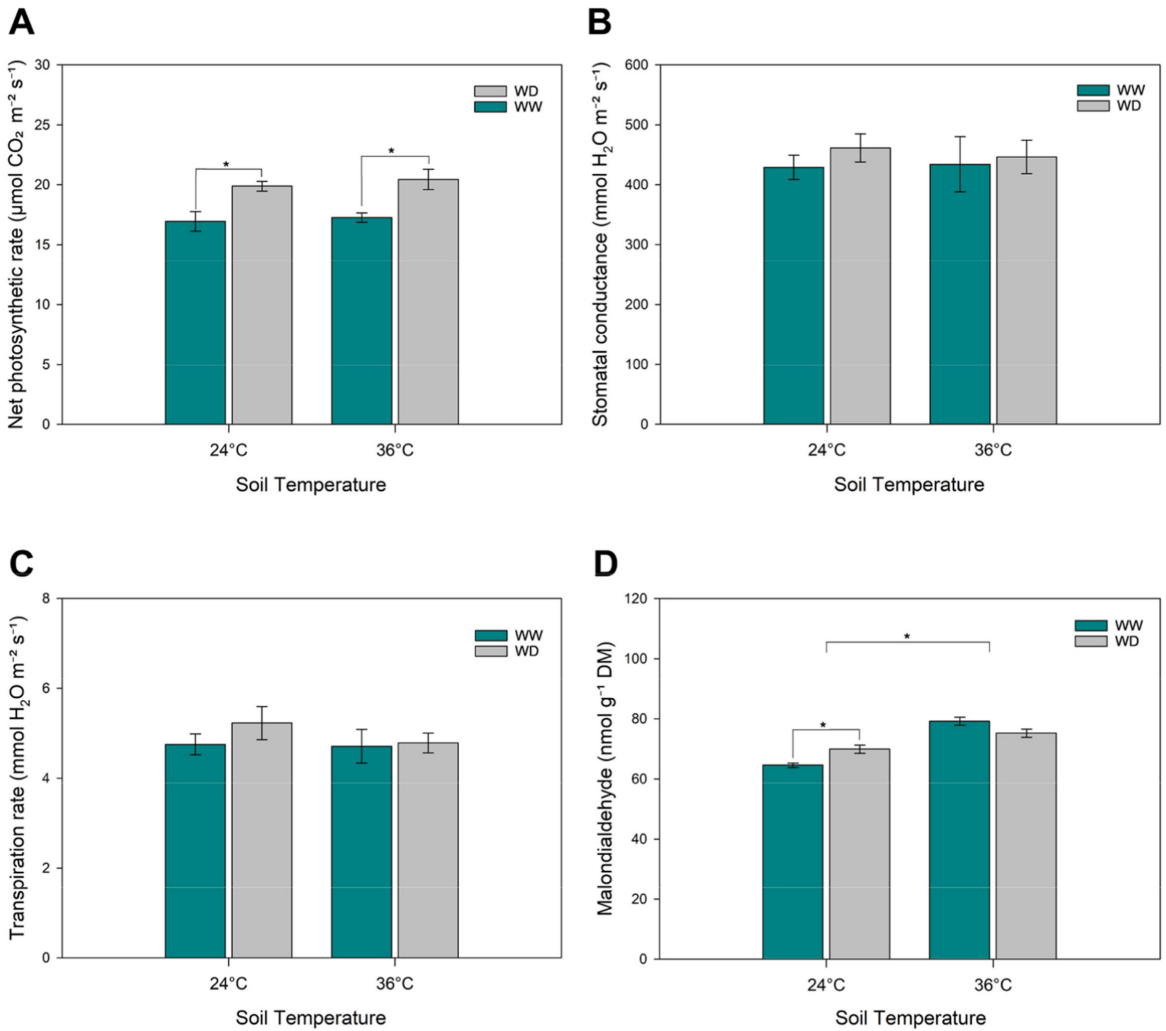
Effects of soil temperatures (24°C and 36°C) and water regimes (WW, well‐watered; WD, water deficit) on gas exchange parameters and oxidative stress indicator. (A) Net photosynthetic rate (μmol CO_2_ m^−2^ s^−1^). (B) Stomatal conductance (mmol H_2_O m^−2^ s^−1^). (C) Transpiration rate (mmol H_2_O m^−2^ s^−1^). (D) Malondialdehyde content (nmol g^−1^ DM). Bars represent mean ± standard error (*n* = 12). Asterisks indicate significant differences between treatments according to Student's *t*‐test (*p* ≤ 0.05).

Physiological parameters associated with gas exchange exhibited differential responses to soil temperature and water availability (Figure 3). The net photosynthetic rate (*A*) was not significantly altered by soil temperature when evaluated independently (24°C vs. 36°C). Conversely, *A* was significantly affected by the water regime at both temperatures (Figure 3A). At 24°C, plants exposed to water deficit showed higher *A* values than well‐watered plants. A comparable response was observed at 36°C, where restricted irrigation also resulted in a significant increase in photosynthetic rate. As a result, *C* assimilation was more strongly influenced by water availability than by soil temperature.

Stomatal conductance (g_*s*_) and transpiration rate (*E*) did not differ significantly across soil temperatures or water regimes (Figure 3B,C). The absence of significant variation in these parameters shows that the changes observed in photosynthesis were not accompanied by detectable stomatal adjustments under the experimental conditions.

Malondialdehyde (MDA) content, an established indicator of lipid peroxidation and oxidative stress, was significantly higher in plants cultivated at 36°C than in those grown at 24°C (Figure 3D), showing enhanced oxidative damage under elevated temperature. At 24°C, water deficit significantly increased MDA levels relative to well‐watered conditions. In contrast, at 36°C, the effect of the water regime was attenuated, with no statistically significant difference observed between well‐watered (WW) and water‐deficit (WD) treatments, showing that elevated soil temperature exerted a more consistent influence on oxidative stress than water limitation alone.

Physiological responses were accompanied by changes in the metabolic profile (Figure S2). Although no statistically significant differences were detected for proline or γ‐aminobutyric acid (GABA) among treatments, proline accumulation tended to be higher under the combined 36°C + WD treatment, whereas GABA accumulation tended to increase under 36°C in well‐watered plants (Figure S3). Heatmap‐based integrative analysis revealed only low correlation coefficients between these metabolites and the integrated BNF indices, indicating a limited functional performance.

Among the organic acids, citrate (CIT) increased significantly at 36°C and showed a positive correlation with the integrated iBNF and ureide concentrations (Figure S3), supporting a close relationship between C metabolism and symbiotic efficiency. Valine and adenosine also responded to soil warming, indicating metabolic reprogramming under elevated temperature. Pyruvate did not exhibit an isolated temperature effect. However, at 36°C, higher concentrations were observed under well‐watered conditions compared with water restriction, indicating that its accumulation was modulated by the interaction between temperature and water availability.

**FIGURE 4:**
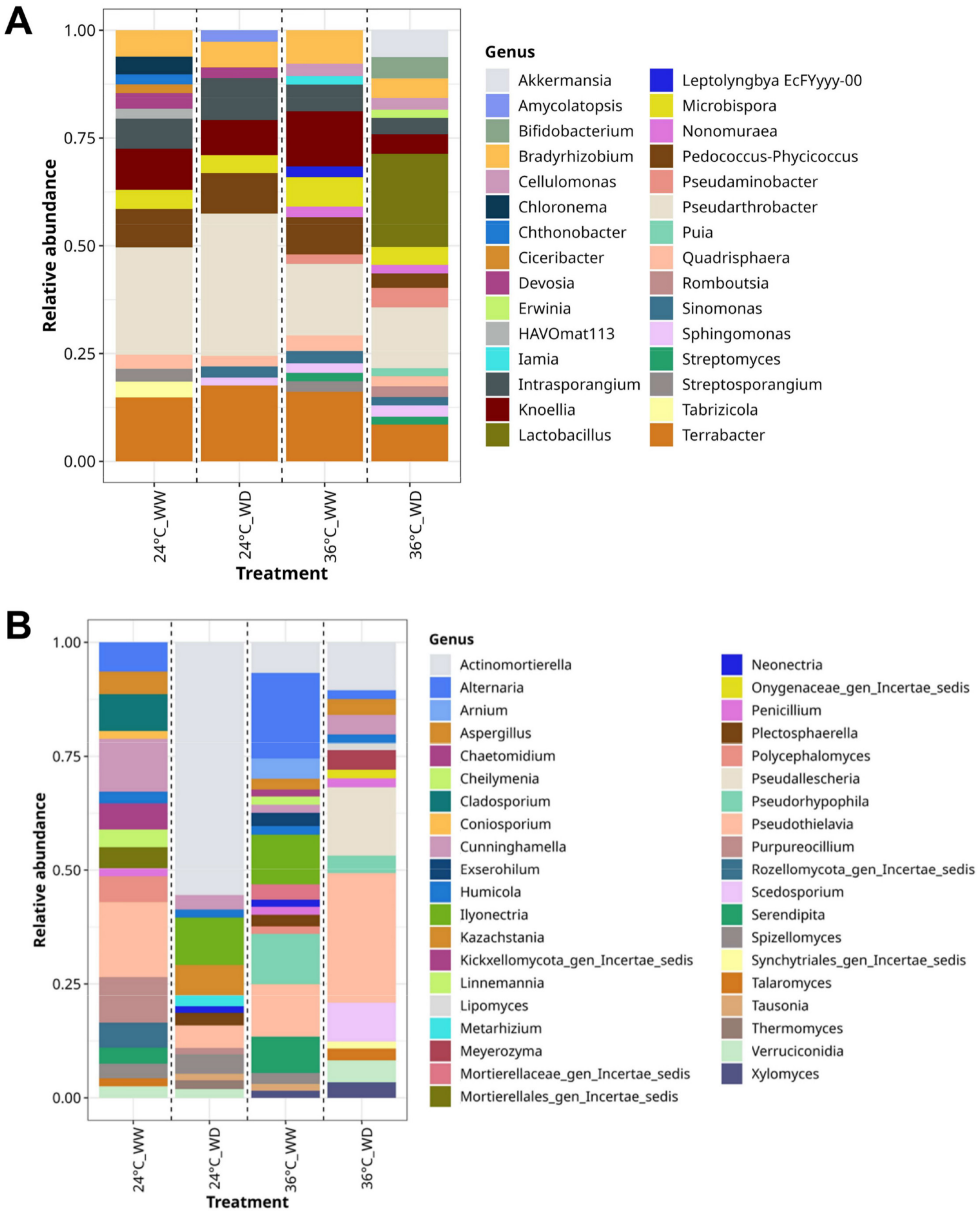
Relative abundance of bacterial (A) and fungal (B) genera across treatments differing in temperature (24°C vs. 36°C) and water regime (well‐watered, WW; water deficit, WD). Stacked bars represent the proportional contribution of each genus to the total community within each treatment. Only genera with > 1% and > 2% relative abundance were included for bacteria and fungi, respectively.

Although gas exchange remained relatively stable in response to variations in soil temperature and water regime, oxidative stress was primarily intensified by soil warming. Concurrently, metabolic reprogramming reflected adjustments in carbon metabolism and redox homeostasis, consistent with changes observed in BNF efficiency (Figures 2 and 4). This indicates the coordinated nature of plant responses to root‐imposed stress.

### 3.4 Integration of Nodule Nutrition With BNF Indices

Variations in BNF efficiency were associated with concomitant changes in the nutritional composition of nodules (Table S2). Soil temperature significantly influenced the majority of macro‐ and micronutrients analyzed. Nodules developed at 36°C showed higher concentrations of N, P, K, S, and B, whereas the concentrations of Ca, Mg, Cu, Fe, Mn, Zn, Ni, Si Co, and Al were greater at 24°C, evidencing strong sensitivity of nodule nutrition to soil warming. In contrast, the effects of the water regime were more specific and temperature‐dependent. At 24°C, only K and Ca differed between well‐watered and water‐restricted plants. At 36°C, water deficit induced additional changes in the concentrations of K, Mg, B, Cu, Zn, and Mo, showing that water limitation intensifies nutritional adjustments when combined with soil warming.

The correlation heatmap between nodule nutrient concentrations and BNF indices further supports this functional integration (Figure S4). Potassium concentration showed a consistent positive correlation with the integrated iBNF and nodule dry mass, whereas Ca, Fe, Mn, and Zn exhibited negative or weak associations. Silicon displayed a persistent negative correlation with BNF‐related variables, indicating a closer association with stress‐adaptive responses rather than with N fixation activity per se.

Indeed, BNF efficiency appears to be linked not only to growth and nodulation, but also to specific changes in nodule nutrient composition. Soil temperature primarily structured these responses, while water availability exerted additional modulation under elevated temperature. These findings align with the multivariate patterns observed in the PCA (Figure 4) and underscore the regulatory role of nodule nutrition in symbiotic performance.

### 3.5 Soil Enzymes in Response to Soil Temperatures and Water Regimes

Soil temperature and water regime significantly affected soil enzyme activities, with responses varying among enzymatic classes (Figure S5). β‐glucosidase activity decreased at 36°C, particularly under well‐watered conditions, whereas water deficit modulated its response under elevated temperature. Arylsulfatase activity was reduced by water restriction at 24°C, while acid phosphatase activity remained relatively stable at 24°C but declined under water deficit at 36°C. In general, soil enzyme activities exhibited temperature and water regime‐dependent responses, indicating differential sensitivity among enzymatic processes to environmental conditions.

### 3.6 Bacterial and Fungal Communities in Response to Soil Temperatures and Water Regimes

Bacterial community composition varied across treatments. While *Terrabacter* and *Pseudarthrobacter* predominated under 24°C conditions, the combined stress of elevated temperature and water deficit (36°C_WD) markedly changed the community, with a strong increase in *Lactobacillus*. Temperature and water availability jointly influenced genus‐level relative abundances, with the most pronounced change under combined stresses (Figure 4A). Fungal communities also responded to the treatments. Under well‐watered conditions, the community showed a relatively even distribution among several genera, whereas drought conditions shifted the community structure toward the predominance of specific taxa (Figure 4B). In particular, *Lipomyces* became more abundant under water regimes, especially at 24°C (24C_WD).

**FIGURE 5:**
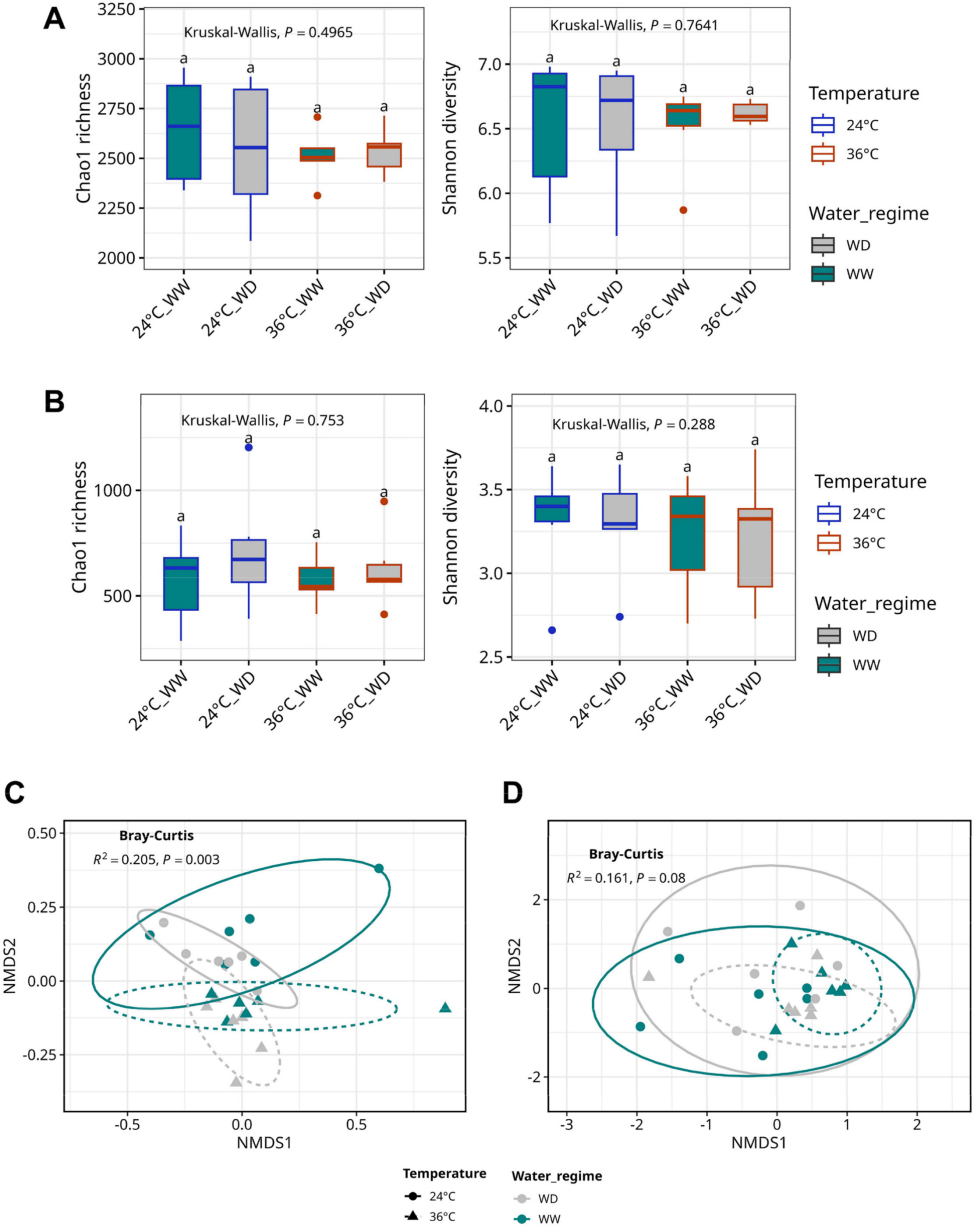
Effects of soil temperatures and water regimes on the diversity and structure of bacterial and fungal communities. Alpha diversity of bacterial (A) and fungal (B) communities under different soil temperatures (24°C vs. 36°C) and water regimes (well‐watered, WW; water deficit, WD). Richness was estimated using the Chao1 index and diversity using the Shannon index. Identical letters indicate the absence of statistical differences (Kruskal–Wallis, *p* > 0.05). Non‐metric multidimensional scaling (NMDS) ordination based on Bray–Curtis dissimilarity showing bacterial (C) and fungal (D) community structure under treatments. Ellipses represent group dispersion.

Despite these compositional shifts, Alpha diversity metrics were not significantly influenced by temperatures or water regimes in either bacterial (A) or fungal (B) communities (Figure 5). Chao1 richness and Shannon diversity showed comparable values across all treatments, with no statistical differences detected (Kruskal–Wallis, *p* > 0.05), indicating overall stability of microbial diversity under the evaluated conditions (Figure 5A–D).

Based on Bray–Curtis dissimilarities, bacterial community composition differed significantly among treatments (PERMANOVA, *R* ^2^ = 0.205, *p* = 0.003), with NMDS ordination indicating that temperature was the main driver of community separation, while water regime had a secondary effect (Figure 5E). In contrast, fungal communities did not differ significantly (*R* ^2^ = 0.161, *p* = 0.08). Overall, bacterial communities appeared more responsive to temperature and water regime, whereas fungal communities showed comparatively greater stability.

Differential abundance analysis revealed several bacterial genera significantly affected by temperature and water deficit, particularly under 36°C conditions (Figure S5). Most bacterial taxa exhibited positive log fold changes (LFC), indicating enrichment under stress treatments, while a few genera (e.g., *Herpetosiphon* and *Actinomycetospora*) showed negative LFC values, suggesting a reduced abundance. Many of these changes were statistically significant (BH‐adjusted *p* ≤ 0.05). For fungi, only two taxa were significantly affected. Both *Purpureocillium* and *Rozellomycota* displayed negative LFC values under 36°C treatments, indicating reduced abundance under elevated soil temperature, with statistically significant differences. In general, bacterial communities showed broader and stronger differential responses to the treatments compared to fungal communities.

Additionally, the bacterial genera *Lysobacter*, *Nitrolancea*, and *Pseudaminobacter* showed the strongest significant correlations with plant stress metabolites (e.g., citrate and proline) and nitrogen‐related traits, suggesting these specific bacteria are the primary drivers of the microbial response to the experimental treatments (Figure S6). Moreover, the bacterial community exhibits higher species richness and core overlap than the fungal community, and while the 24°C_WD condition uniquely hosts the highest diversity for both groups (Figure S6). Rarefaction curves confirm that sequencing depth was sufficient to capture representative diversity across all samples (Figure S7).

### 3.7 Multivariate Analysis of Growth, Physiology, Metabolism, and BNF Responses

**FIGURE 6:**
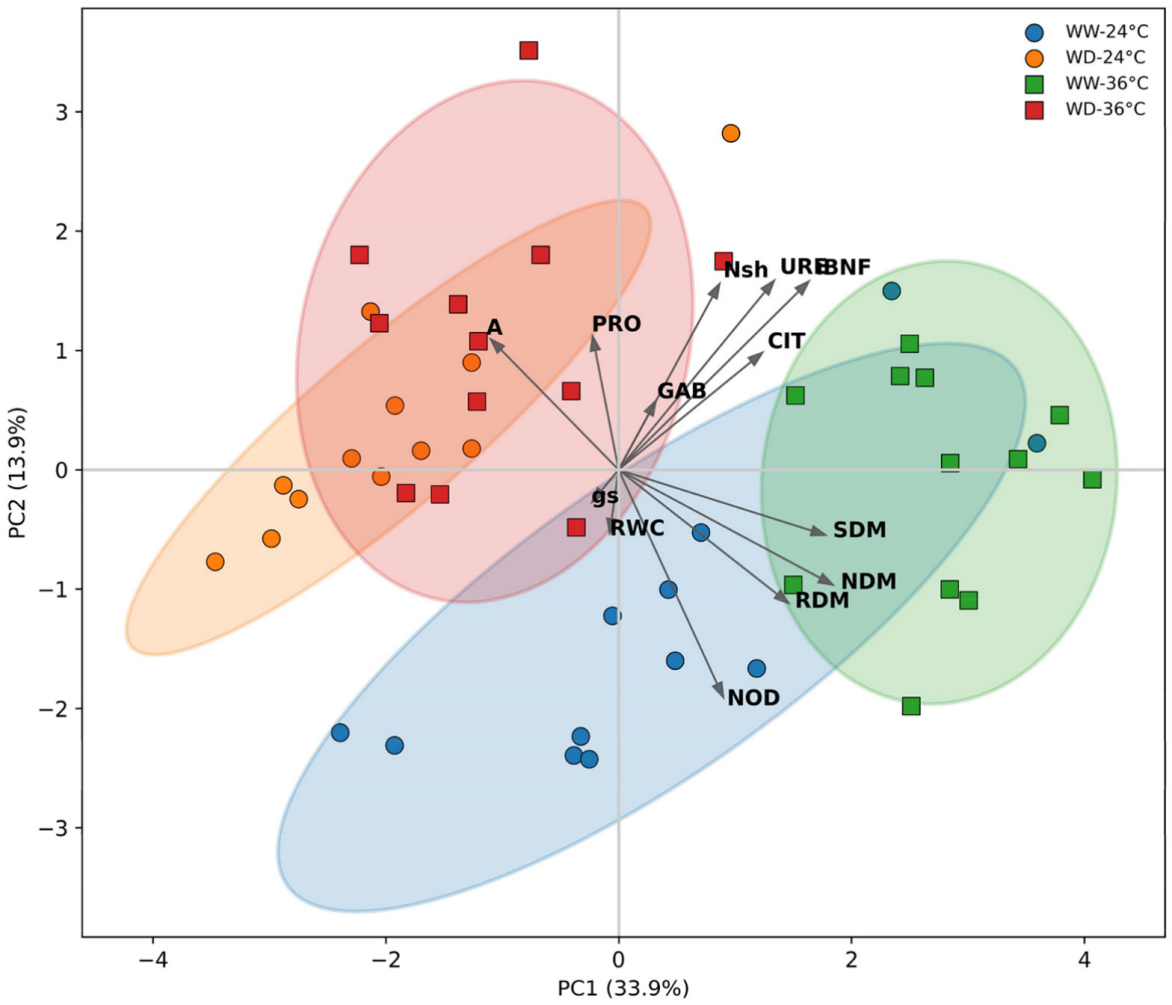
Principal component analysis (PCA) of physiological, nodulation, and metabolic variables in soybeans under different treatments. Data were standardized (*Z*‐score) prior to analysis. PC1 and PC2 explained 33.9% and 13.9% of the total variance, respectively. Symbols represent individual replicates, ellipses indicate treatment clustering (WW‐24°C, WD‐24°C, WW‐36°C, WD‐36°C), and vectors indicate variable loadings. A, net photosynthetic rate; CIT, citrate; GAB = GABA, γ‐aminobutyric acid; gs, stomatal conductance; iBNF, BNF index; NDM, nodule dry mass; NOD, nodule number; Nsh, shoot nitrogen concentration; PRO, proline; RDM, root dry mass; RWC, relative water content; SDM, shoot dry mass; URE, ureide concentration; WD, water deficit; WW, well‐watered.

Principal component analysis (PCA) was performed to integrate 13 variables related to plant growth, leaf physiology, metabolism, and BNF efficiency, providing a comprehensive assessment of plant responses to soil temperatures and water regimes (Figure 6). The analyzed variables included the integrated iBNF, ureide concentration (URE), nodule dry mass (NDM), nodule number (NOD), shoot nitrogen content (Nsh), shoot dry mass (SDM), root dry mass (RDM), relative water content (RWC), proline (PRO), net photosynthetic rate (*A*), stomatal conductance (*g*_*s*_), citrate (CIT), and γ‐aminobutyric acid (GABA).

The first two principal components accounted for a substantial proportion of the total variance and enabled a clear discrimination among treatments according to water availability and, to a lesser extent, soil temperature. Principal component 1 (PC1) was strongly associated with variables directly related to plant growth and BNF performance, including iBNF, URE, NDM, SDM, Nsh, and RDM, as well as NOD. These variables exhibited high positive loadings, indicating that treatments positioned positively along PC1 were characterized by enhanced growth, greater nodulation, and improved overall BNF efficiency.

Treatments under well‐watered conditions were predominantly clustered in the positive region of PC1, reflecting the higher biomass accumulation, enhanced nodulation, greater shoot nitrogen content, and elevated BNF indices observed in the univariate analyses. In contrast, plants subjected to water deficit were associated with negative PC1 scores, consistent with the significant reductions in shoot and root dry mass, nodule biomass, integrated iBNF, and ureide. Overall, the water regime represented the primary structuring factor of the multivariate space, whereas soil temperature modulated responses within each water condition.

The second principal component (PC2) was primarily associated with physiological and metabolic variables, including *A*, PRO, and to a lesser extent, GABA and CIT. Among these, *A* contributed most strongly to the composition of this axis, whereas RWC and *g*_*s*_ exhibited comparatively lower loadings. Although the PCA identified a clear physiological gradient among treatments, univariate analyses indicated that only *A* differed significantly, whereas RWC, *g*_*s*_, and transpiration rate (*E*) did not show significant variation across treatments.

Among the analyzed metabolites, citrate (CIT) occupied an intermediate position in the multivariate space, showing a closer association with growth and BNF‐related variables than with canonical stress‐associated metabolites. This distribution supports its integrative role in linking carbon metabolism to symbiotic performance and is consistent with the positive correlations observed between CIT, iBNF, and ureide levels in the supplementary analyses.

The PCA results demonstrate a strong coupling between BNF efficiency, plant growth, and nodulation. Physiological and metabolic variables substantially structured the multivariate space, with photosynthetic rate and carbon‐related metabolites exhibiting the strongest contributions. The separation of treatments along the principal axes indicates that soil temperature and water availability coordinately modulated growth, metabolism, and BNF, highlighting integrated physiological adjustments among treatments.

**FIGURE 7:**
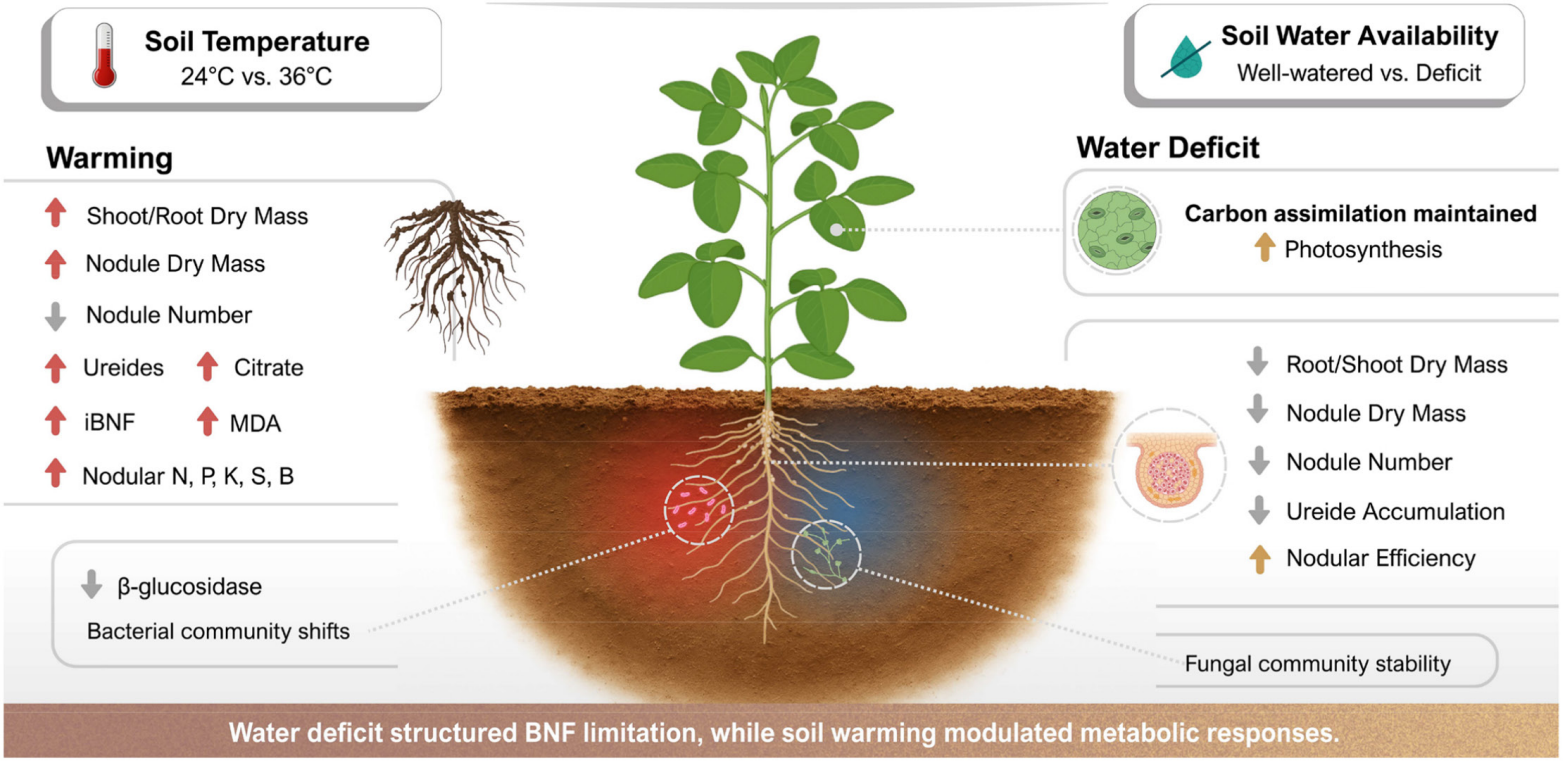
Summary of the effects of soil warming and water deficit on soybean growth, nodulation, biological nitrogen fixation (BNF), metabolism, nutrition, and soil microbial communities. Soil warming promoted plant growth, nodule development, BNF performance, metabolic and nutritional adjustments, and bacterial community shifts, whereas water deficit primarily reduced plant growth, nodulation, and BNF, despite maintaining carbon assimilation and increasing nodular efficiency. This indicates that water deficit was the main driver of BNF limitation, while soil warming enhanced symbiotic performance and predominantly modulated metabolic, nutritional, and rhizosphere microbial responses.

Overall, our findings showed that soil warming (36°C) increased shoot/root dry mass, nodule dry mass, ureides, iBNF, citrate, MDA, and nodular nutrient accumulation (N, P, K, S, and B), while reducing nodule number and β‐glucosidase activity, accompanied by shifts in bacterial community structure. Water deficit reduced root/shoot dry mass, nodule dry mass, nodule number, and ureide accumulation, although carbon assimilation (photosynthesis) was maintained and nodular efficiency increased under stress conditions. In addition, fungal community stability was preserved under water deficit (Figure 7). Moreover, water deficit primarily constrained BNF, whereas soil warming modulated metabolic and microbial responses.

## 4 Discussion

### 4.1 Root‐Zone Warming as a Positive Modulator of Symbiosis Under Adequate Water Availability

Soil temperature is a key regulator of nodulation. Elevated temperatures commonly restrict nodule initiation and nitrogenase activity (Zahran 1999; Hungria and Vargas 2000; Ramírez et al. 2023; Yeremko et al. 2025), whereas moderate increases may stimulate nodule development depending on host–rhizobium compatibility (Munévar and Wollum 1981). In the present study, root‐zone warming to 36°C under well‐watered conditions did not impair the soybean–rhizobium symbiosis; instead, it enhanced nodule growth, functional performance, and overall BNF efficiency. These results demonstrate that the effects of soil temperatures on BNF are strongly context‐dependent and mediated by water availability. The increase in BNF efficiency suggests that moderate warming promoted functional adjustments in the rhizosphere (Zhao et al. 2023), strengthening metabolic coordination between *Bradyrhizobium* and the host plant through regulated molecular signaling processes characteristic of the rhizobium–legume symbiosis (Buhian and Bensmihen 2018).

Although nodule number declined at 36°C, the increase in individual nodule mass and activity indicates a qualitative shift in symbiotic regulation within a physiologically tolerable thermal range. This adjustment is consistent with activation of the autoregulation of nodulation (AON) pathway (Gresshoff et al. 2025), which restricts auxin transport and suppresses Nod‐factor signaling, limiting nodule initiation (Reid et al. 2012; Ryu et al. 2012; Pervent et al. 2021). Through modulation of the nodule number, the plant likely minimized excessive carbon costs (Chaulagain and Frugoli 2021), while sustaining effective organogenesis (Reid et al. 2017; Fisher et al. 2018) and ensuring sufficient N_2_‐fixing tissues (King and Purcell 2001). Adequate water and carbohydrate availability supported nodule metabolism and ureide export, contributing to functional stability under warming.

The maintenance of photosynthetic assimilation under isolated warming reinforces these findings, since carbon limitation is a major constraint on BNF under stress (Ashraf and Harris 2013; Mcculloch et al. 2026; Gupta et al. 2026). Symbiosis depends on photosynthesis‐derived sucrose supplied to nodules (Lepetit and Brouquisse 2023; Luan et al. 2025), and the preservation of the photoassimilate flow likely allowed the elevated soil temperature to act primarily as a metabolic stimulus rather than a limiting factor.

Although oxidative markers increased (e.g., MDA and proline accumulation), the absence of functional impairment indicates that redox changes remained within adaptive thresholds. Depending on intensity, warm temperatures can trigger thermotolerance and acclimation responses (Kan et al. 2023). Enhanced metabolic activity may have activated stress signaling pathways (Farmer and Mueller 2013; Jumrani and Bhatia 2019; Ahammed et al. 2024) alongside antioxidant defenses and proline‐associated responses that support osmotic and cellular adjustments (Moloi and van der Merwe 2021; Song et al. 2022), reflecting regulated redox balance rather than oxidative damage per se (Imran et al. 2021; Zhang et al. 2021).

Thermal effects were not restricted to roots, but extended to the shoots, indicating coordinated whole‐plant responses within the symbiotic unit (Peng et al. 2025). Moderately elevated temperatures may promote thermomorphogenesis (Quint et al. 2016) through hormonal regulation that stimulates cell division and expansion (Gray et al. 1998; Li et al. 2022), supporting biomass accumulation and source–sink balance (Kang et al. 2024). These responses reinforce the role of roots as primary sensors of soil temperature fluctuations capable of generating systemic growth signals (Gul et al. 2026).

Higher ureide concentrations under 36°C + WW further indicate sustained or enhanced nodular activity. Under favorable energy and water supply, nodules maintain high nitrogenase activity, assimilate NH_4_^+^ into glutamine, and channel it through the purine pathway for ureide synthesis (Schubert 1986; Taiz et al. 2017). Thus, increased ureides reflect efficient nitrogen export from roots to shoots (Sinclair and Serraj 1995; Cerezini et al. 2017). Besides acting as the principal transport form of fixed nitrogen in soybeans, ureides also participate in root–shoot signaling that regulates BNF according to the plants' metabolic demands (Baral et al. 2016; Thu and Tegeder 2025). In particular, allantoine may exert regulatory functions under stress by modulating tolerance‐related responses (Lescano et al. 2016; Kaur et al. 2021, 2023). Therefore, ureide accumulation under warming with adequate water supply reflects not only maintained BNF but also efficient integration among nodule metabolism, nitrogen transport, and systemic signaling.

Overall, these results indicate that the effects of soil temperature on symbiosis are strongly conditioned by water availability. In the absence of water limitation, moderate soil warming can stimulate nodular metabolic activity and functional efficiency, challenging the generalized assumption that elevated temperatures inherently impair BNF.

#### 4.1.1 Nodular Nutritional Reconfiguration Under Root Warming

Modifications in nodule nutrient composition indicate that the efficiency of BNF was closely linked to metabolic reorganization triggered by root warming. The elevated concentrations of N, P, and K observed at 36°C suggest increased bioenergetic and metabolic demands. Phosphorus plays a pivotal role in ATP synthesis, thereby sustaining nitrogenase activity and nodule development (Lu et al. 2020; Zhong et al. 2023), while potassium contributes to enzymatic activation and the maintenance of redox homeostasis under stress conditions (Xia et al. 2023; Ma et al. 2025). The higher nodular N content may further reflect feedback regulatory mechanisms determining nodulation and nitrogen assimilation (Lin et al. 2018).

Concomitantly, the increase in S is consistent with its structural and redox functions in proteins essential for symbiosis and antioxidant activity (Krishnan et al. 2018; Johansson et al. 2025), whereas B may contribute to the structural stability of nodular tissues during infection and cell wall organization (Bolaños et al. 1996; Garrone et al. 2025). Thus, symbiotic efficiency under soil warming appears to depend more strongly on internal nutritional configuration than solely on nodule number.

### 4.2 Water Restriction as the Primary Structural Constraint on Nodulation and BNF

Among the tested factors, water restriction had the most pronounced impact on nodulation, resulting in substantial losses in nodule biomass and a corresponding decrease in total BNF. This decline corroborates previous evidence that water availability is essential for the establishment and maintenance of functional nodules (Djekoun and Planchon 1991; Mastrodomenico et al. 2013; Lumactud et al. 2023). Water deficit interferes with the initial stages of symbiosis, disrupting rhizobial chemotaxis and root signaling processes, and consequently limiting infection and root hair development (Buhian and Bensmihen 2018; Chakraborty et al. 2022; Lin et al. 2023). It also accelerates nodule senescence and compromises symbiotic tissue integrity, restricting nitrogenase activity and nitrogen export (Ruiz‐Lozano et al. 2001; Dhanushkodi et al. 2018; Kazmierczak et al. 2020).

In addition to these structural effects, water deficit reshapes source–sink relationships. Because nodules represent carbon‐ and water‐intensive sinks, plants reallocate photoassimilates under drought to sustain cellular integrity and shoot function (Miyoshi et al. 2025). Isotopic (^13^C) tracing in N_2_‐fixing soybean demonstrates that water limitation alters carbon partitioning among shoots, roots, and nodules (Rubia et al. 2025), while the reduced sink strength of nodules under drought has been shown to limit their capacity to utilize photoassimilates (Parvin et al. 2020). Hence, reductions in BNF are driven not only by lower photosynthetic supply, but also by constraints in carbon distribution and metabolic capacity within roots and nodules.

Because specific nodular efficiency reflects nitrogen accumulation on a per‐nodule basis, the reduction in nodulation likely reflects a regulatory adjustment in carbon distribution, favoring the maintenance of fewer but metabolically efficient nodules (Tasca et al. 2024). Autoregulation of nodulation (AON) may further modulate this response, aligning nodule number with plant physiological status under water deficit (Kawade et al. 2024). This reallocation may support the continued activity of the remaining nodules, which could account for the observed increases in both nodular efficiency and specific nodular efficiency despite reduced nodulation.

Consistent with the proposed mechanism, reduced ureide content and integrated BNF values point to decreased total nitrogen contribution under water deficit, while higher specific nodular efficiency indicates that the remaining nodules sustained elevated metabolic activity. Symbiotic nitrogen fixation depends not merely on photosynthetic carbon supply, but on the efficient partitioning of assimilates to nodules and their internal metabolic functionality (Aranjuelo et al. 2014). Classical studies further indicate that limitations may arise at the level of transport or nodule metabolism even when carbon assimilation remains high (Williams et al. 1982).

Notably, the lack of additional impairment under combined soil warming and drought, despite maintained photosynthetic rates, indicates functional reorganization rather than additive stress effects. Unlike reports of reduced carbon assimilation under concurrent heat and drought (Suzuki et al. 2014; Cohen et al. 2021), leaf assimilation remained stable, indicating that BNF limitation was primarily associated with altered carbon allocation and nodular metabolic capacity.

Evidence from metabolic profiling corroborates these observations. Changes in citrate, adenosine, and pyruvate concentrations indicate a shift in carbon flux at the interface between glycolysis and the tricarboxylic acid (TCA) cycle (Sweetlove et al. 2010; Zheng et al. 2025), suggesting respiratory reprogramming rather than a typical osmotic stress response. The accumulation of citrate is consistent with its reported role in enhancing abiotic stress tolerance through modulation of enzymatic activity and osmotic balance (Tahjib‐Ul‐arif et al. 2021; Zhang and Fernie 2023). Moreover, the stability of proline and γ‐aminobutyric acid (GABA) levels indicates the absence of severe metabolic collapse (Signorelli et al. 2021). These trends suggest that energetic adjustment, potentially sustained by maintained TCA cycle activity and GABA‐mediated coordination of carbon and nitrogen metabolism (Fait et al. 2008; Hasan et al. 2021), contributed to the partial preservation of nodular functionality.

Overall, these findings demonstrate that drought‐induced nodulation decline represents an adaptive regulatory adjustment of carbon–nitrogen interactions, with lower total nodule biomass but improved specific fixation efficiency in the residual nodular population.

### 4.3 Microbial Structure and Functional Implications

Consistent with broader evidence from climate‐change research, our results demonstrate that edaphic stressors such as soil warming and water deficit primarily restructure bacterial community composition rather than simply reducing overall alpha diversity. Many studies show that elevated temperatures and reduced moisture drive taxonomic turnover in soil bacterial assemblages, altering beta diversity without necessarily decreasing alpha diversity, a pattern attributed to environmental filtering favoring stress‐tolerant taxa over sensitive groups (Zhao et al. 2024).

The stronger response of bacterial communities relative to fungal assemblages aligns with findings from mesocosm experiments showing that drought destabilizes bacterial networks more than fungal networks, likely due to differences in resilience and life‐history strategies between prokaryotes and fungal eukaryotes (de Vries et al. 2018). Under combined warming and drought, changes toward taxa capable of withstanding abiotic stress (e.g., Gram‐positive groups like Actinobacteriota) have been repeatedly documented, suggesting that microbiomes may reorganize toward drought‐adapted communities under more extreme conditions (De Silva et al. 2025).

Correlation analyses linking specific bacterial taxa with plant nitrogen traits and stress metabolites reflect an emerging understanding of how stress‐responsive microbes can influence nutrient cycling and host interactions. Plant growth‐promoting bacteria, for instance, often exhibit enhanced functional potential under drought, including nutrient solubilization and nitrogen‐cycling activities that can modulate plant responses to water limitation (De Silva et al. 2025).

In contrast, fungal communities in many agricultural and grassland soils exhibit greater structural stability under short‐term drought and thermal fluctuations, often maintaining alpha diversity despite compositional changes (Cheng et al. 2017; De Silva et al. 2025). This resilience may reflect the fungi's extensive hyphal networks and physiological strategies (e.g., osmolyte accumulation) that confer resistance to moisture and temperature extremes.

Functional indicators, including soil enzyme activities, further support the notion that microbial nutrient cycling processes are regulated by climatic stressors. Abiotic stress conditions alter carbon, phosphorus, and sulfur cycling, likely through changes in microbial community structure and physiological responses (De Silva et al. 2025). Therefore, climate‐related stressors may drive bacterial community restructuring, with potential downstream consequences for nutrient transformations and plant–microbe interactions, whereas fungal assemblages exhibit comparatively greater structural resilience under the stress conditions examined here.

## 5 Conclusions

This study demonstrates that biological nitrogen fixation in soybeans is regulated by the interaction between soil temperature and water availability, with the experimental design including individual stress conditions (24°C + WD and 36°C + WW), the combined stress condition (36°C + WD), and the reference control treatment (24°C + WW), allowing direct comparisons among the isolated and interacting effects of soil warming and water deficit. Water deficit acted as the main structural constraint on symbiosis, markedly reducing nodulation and the overall contribution of BNF. In contrast, moderate root‐zone warming under adequate water supply promoted plant growth and symbiotic performance, indicating that elevated soil temperature does not necessarily impair BNF efficiency. Physiological and metabolic responses suggest that the maintenance of carbon metabolism plays a central role in sustaining nodular activity even when structural changes in nodulation occur. In addition, shifts in rhizosphere bacterial communities indicate that symbiotic responses are accompanied by adjustments in the soil microbiome. Ultimately, these findings highlight the root system as a central component integrating thermal and hydric signals that regulate symbiotic functioning and biological nitrogen fixation in soybeans. Understanding the interaction between soil temperature and water availability is therefore essential for predicting soybean performance under future climate change scenarios.

## Author Contributions

**Camila Domingos Cabral:** conceptualization, methodology, formal analysis, investigation, data curation, writing – original draft, writing – review and editing. **Gladys Angélica Apaza‐Castillo:** methodology, formal analysis, investigation, data curation. **Adriana Sturion Lorenzi:** investigation, writing – original draft, writing – review and editing draft. **Pedro Henrique Pedron Mattiuzzi:** methodology, formal analysis, investigation, data curation. **Anaila Amaral de Alencar:** methodology, formal analysis. **Maria Carolina Quecine:** investigation, validation, writing – review and editing. **Moacir Tuzzin de Moraes:** investigation, validation, writing – review and editing. **Flávio Henrique Silveira Rabêlo:** writing – review and editing. **Paulo Mazzafera:** writing – review and editing. **Tiago Tezotto:** conceptualization, methodology, formal analysis, investigation, data curation, validation, supervision, writing – original draft, writing – review and editing.

## Funding

This work was supported by Coordenação de Aperfeiçoamento de Pessoal de Nível Superior, PROEX Program (Process No. 88887.906771/2023‐00), Finance Code 001, Project numbers 601531 and 601461; Fundação de Estudos e Pesquisas Agrícolas e Florestais, Institutional Scholarship Program (BES, No. 1185 PIB); Fundação de Apoio à Univesidade de São Paulo, InnovaPower TotalEnergies Program (Process No. 7867).

## Conflicts of Interest

The authors declare no conflicts of interest.

## Supporting information


**Table S1:** Chemical and granulometric characterization of the soil used in the experiment.
**Table S2:** Mineral composition of soybean nodules as affected by soil temperature and water regime.
**Figure S1:** Effects of soil temperatures (24°C and 36°C) and water regimes (WW, well watered; WD, water deficit) on shoot nitrogen status, ureide concentration, and leaf water relations. (A) Shoot nitrogen concentration (g kg⁻1 DM). (B) Shoot nitrogen accumulation (mg N plant⁻1). (C) Ureide concentration in shoot dry matter (mg kg⁻1 DM). (D) Leaf relative water content (%). Bars represent mean ± standard error (*n* = 12). Asterisks indicate significant differences between treatments according to Student's t‐test (*p* ≤ 0.05).
**Figure S2:** Integrated correlation matrix between metabolites and biological nitrogen fixation (BNF)‐related variables in soybean plants. The color scale represents Pearson's correlation coefficient (r), ranging from −1 to +1, where positive values indicate direct correlations and negative values indicate inverse correlations.
**Figure S3:** Effects of treatments on metabolite concentration in soybean. (A) Citrate. (B) Pyruvate. (C) Valine. (D) Adenosine. (E) GABA. (F) Proline. WW, well‐watered; WD, water deficit. Values represent mean ± standard error (*n* = 12). Asterisks indicate significant differences between treatments according to Student's t‐test (*p* ≤ 0.05).
**Figure S4:** Correlation matrix between nodular nutrients and BNF indices in soybean. Correlations were calculated using Pearson's correlation coefficient (r). The color scale ranges from −1 (negative correlation) to +1 (positive correlation), indicating the strength and direction of associations. iBNF, integrated BNF efficiency index; URE, ureides; NDM, nodule dry mass.
**Figure S5:** Effects of treatments on soil enzyme activities associated with nutrient cycling. (A) β‐glucosidase. (B) Arylsulfatase. (C) Acid phosphatase. WW, well‐watered; WD, water deficit. Values represent mean ± standard error (*n* = 12). Asterisks indicate significant differences between treatments according to Student's t‐test (*p* ≤ 0.05).
**Figure S6:** Differential abundance of bacterial (A) and fungal (B) taxa across treatments (24°C_WD, 36°C_WW, and 36°C_WD), using 24°C_WW as the control. Taxa responses are expressed as log fold change (LFC), indicating enrichment or depletion relative to the reference condition, with statistical significance determined by adjusted *p*‐values. (C) Correlation network and matrix illustrating relationships among soil chemical attributes and dominant bacterial genera. Correlations are based on Pearson's coefficients, and significant associations are further supported by Mantel test results, where edge thickness reflects correlation strength and color denotes statistical significance.
**Figure S7:** Analysis of microbial community richness and sampling depth. UpSet plots illustrating the number of shared and unique bacterial (A) and fungal (B) species across the different temperature and water treatments (24°C_WD, 36°C_WW, 36°C_WD, and the 24°C_WW control). Vertical bars represent the intersection size of species sets, while the matrix below indicates the specific treatment combinations compared. Rarefaction curves for bacterial (C) and fungal (D) communities showing the number of observed species as a function of the number of sequences (sample size).

## Data Availability

The data that support the findings of this study are openly available in European Nucleotide Archive (ENA) at https://www.ebi.ac.uk/ena/browser/home, reference number PRJEB108728 and PRJEB108779.
