# Supplementary material for: Temperature‐Dependent Root Responses to Water Deficit Modulate Biological Nitrogen Fixation and Rhizosphere Dynamics in Soybeans
Source: Physiol Plant. 2026 Jul 22;178(4):e71029. doi: 10.1111/ppl.71029 (PMC13390661; doi:10.1111/ppl.71029)
Supplement: Supplementary file 1 — Table S1: Chemical and granulometric characterization of the soil used in the experiment. Table S2: Mineral composition of soybean nodules as affected by soil temperature and water regime. Figure S1: Effects of soil temperatures (24°C and 36°C) and water regimes (WW, well watered; WD, water deficit) on shoot nitrogen status, ureide concentration, and leaf water relations. (A) Shoot nitrogen concentration (g kg⁻1 DM). (B) Shoot nitrogen accumulation (mg N plant⁻1). (C) Ureide concentration in shoot dry matter (mg kg⁻1 DM). (D) Leaf relative water content (%). Bars represent mean ± standard error (n = 12). Asterisks indicate significant differences between treatments according to Student's t‐test (p ≤ 0.05). Figure S2: Integrated correlation matrix between metabolites and biological nitrogen fixation (BNF)‐related variables in soybean plants. The color scale represents Pearson's correlation coefficient (r), ranging from −1 to +1, where positive values indicate direct correlations and negative values indicate inverse correlations. Figure S3: Effects of treatments on metabolite concentration in soybean. (A) Citrate. (B) Pyruvate. (C) Valine. (D) Adenosine. (E) GABA. (F) Proline. WW, well‐watered; WD, water deficit. Values represent mean ± standard error (n = 12). Asterisks indicate significant differences between treatments according to Student's t‐test (p ≤ 0.05). Figure S4: Correlation matrix between nodular nutrients and BNF indices in soybean. Correlations were calculated using Pearson's correlation coefficient (r). The color scale ranges from −1 (negative correlation) to +1 (positive correlation), indicating the strength and direction of associations. iBNF, integrated BNF efficiency index; URE, ureides; NDM, nodule dry mass. Figure S5: Effects of treatments on soil enzyme activities associated with nutrient cycling. (A) β‐glucosidase. (B) Arylsulfatase. (C) Acid phosphatase. WW, well‐watered; WD, water deficit. Values represent mean ± standard error (n = 12). Aster [file PPL-178-e71029-s001.pdf]

Temperature-dependent root responses to water deficit modulate biological nitrogen fixation and rhizosphere dynamics in soybeans

Camila Domingos Cabral [1], Gladys Angélica Apaza-Castillo [2,3], Adriana Sturion Lorenzi [1], Pedro Henrique Pedron Mattiuzzi [2,3], Anaila Amaral de Alencar [1], Maria Carolina Quecine [3], Moacir Tuzzin de Moraes [1], Flávio Henrique Silveira Rabêlo [1], Paulo Mazzafera [4,5] and Tiago Tezotto[1*]

[1] Department of Soil Science, "Luiz de Queiroz" College of Agriculture (Esalq), University of São Paulo (USP), Piracicaba 13418-900, São Paulo, Brazil

[2] Center for Nuclear Energy in Agriculture (CENA), University of São Paulo (USP), Piracicaba 13416-000, São Paulo, Brazil

[3] Department of Genetics, "Luiz de Queiroz" College of Agriculture (Esalq), University of São Paulo (USP), Piracicaba 13418-900, São Paulo, Brazil

[4] Department of Biology, Institute of Biology, State University of Campinas (UNICAMP), Campinas 13083-970, São Paulo, Brazil

[5]Institute of Science and Technology, Federal University of São Paulo (UNIFESP), São José dos Campos 12247-014, São Paulo, Brazil

Correspondence:
*Corresponding author,
E-mail: tiago.tezotto@usp.br (T.T.)

Supplementary Tables:

Suppl. Table S1. Chemical and granulometric characterization of the soil used in the experiment.

Parameter	Unit	Measured value
**Macronutrients**		
Phosphorus (P)	mg dm^{-3}	3.30
Sulfur (S)	mg dm^{-3}	9.00
Potassium (K^+)	$mmol_c$ dm^{-3}	0.40
Calcium (Ca^{2+})	$mmol_c$ dm^{-3}	9.10
Magnesium (Mg^{2+})	$mmol_c$ dm^{-3}	4.00
**Micronutrients**		
Boron (B)	mg dm^{-3}	0.24
Copper (Cu^{2+})	mg dm^{-3}	0.50
Iron (Fe)	mg dm^{-3}	34.60
Manganese (Mn^{2+})	mg dm^{-3}	4.60
Zinc (Zn^{2+})	mg dm^{-3}	0.70
**General chemical properties**		
pH ($CaCl_2$)	-	4.51
Organic Matter (OM)	g dm^{-3}	9.40
Aluminum (Al^{3+})	$mmol_c$ dm^{-3}	1.40
Potential acidity (H+Al)	$mmol_c$ dm^{-3}	23.40
Sum of bases (SB)	$mmol_c$ dm^{-3}	13.50
Cation exchange capacity (CEC)	$mmol_c$ dm^{-3}	36.90
Base saturation (V)	%	37.00
Aluminum saturation (m)	%	9.00
**Physical properties**		
Sand	g kg^{-1}	797.00
Silt	g kg^{-1}	28.00
Clay	g kg^{-1}	175.00

Element extraction and soil property determination: P and K extracted by resin; S by 0.01 mol L^{-1} calcium phosphate; Ca, Mg, and Al by 1 mol L^{-1} KCl; Cu, Fe, Mn, and Zn extracted with DTPA (diethylenetriaminepentaacetic acid) and

analyzed by atomic absorption spectrophotometry; B extracted with hot water and determined colorimetrically; pH measured in $CaCl_2$; OM determined colorimetrically; total sand = 2 to 0.05 mm; silt = 0.05 to 0.002 mm; clay dispersed <0.002 mm.

Suppl. Table S2. Mineral composition of soybean nodules as affected by soil temperature and water regime.

Nutrient	Temperature		Water regime within temperature			
	24 °C	36 °C	24 °C WW	24 °C WD	36 °C WW	36 °C WD
N (g kg⁻¹)	43.28 ± 0.69 B	46.29 ± 0.65 A	43.05 ± 1.07 a	43.51 ± 0.90 a	46.56 ± 0.63 a	46.03 ± 1.17 a
P (g kg⁻¹)	3.92 ± 0.12 B	4.27 ± 0.12 A	3.95 ± 0.15 a	3.89 ± 0.18 a	4.40 ± 0.21 a	4.14 ± 0.12 a
K (g kg⁻¹)	2.57 ± 0.11 B	3.12 ± 0.14 A	2.91 ± 0.15 a	2.22 ± 0.09 b	3.65 ± 0.15 a	2.58 ± 0.10 b
Ca (g kg⁻¹)	3.50 ± 0.09 A	3.14 ± 0.07 B	3.31 ± 0.11 b	3.69 ± 0.13 a	3.07 ± 0.10 a	3.21 ± 0.09 a
Mg (g kg⁻¹)	1.81 ± 0.05 A	1.68 ± 0.04 B	1.88 ± 0.07 a	1.75 ± 0.06 a	1.80 ± 0.06 a	1.56 ± 0.05 b
S (g kg⁻¹)	2.35 ± 0.05 B	2.53 ± 0.05 A	2.33 ± 0.06 a	2.37 ± 0.09 a	2.49 ± 0.07 a	2.57 ± 0.06 a
B (mg kg⁻¹)	13.48 ± 0.94 B	19.48 ± 1.43 A	13.76 ± 1.69 a	13.21 ± 0.91 a	16.68 ± 1.76 b	22.29 ± 2.00 a
Cu (mg kg⁻¹)	10.77 ± 0.79 A	8.07 ± 0.24 B	11.49 ± 1.45 a	10.05 ± 0.62 a	7.22 ± 0.21 b	8.92 ± 0.26 a
Fe (mg kg⁻¹)	1137 ± 69.9 A	911 ± 39.3 B	1062 ± 76.1 a	1212 ± 116.9 a	855 ± 46.4 a	967 ± 61.0 a
Mn (mg kg⁻¹)	12.72 ± 0.81 A	10.44 ± 0.51 B	12.32 ± 1.00 a	13.12 ± 1.30 a	10.02 ± 0.62 a	10.85 ± 0.81 a
Zn (mg kg⁻¹)	32.74 ± 1.47 A	24.12 ± 0.62 B	34.48 ± 2.52 a	31.01 ± 1.45 a	22.50 ± 0.82 b	25.75 ± 0.65 a
Mo (mg kg⁻¹)	15.01 ± 1.01 A	13.11 ± 0.56 A	16.84 ± 1.65 a	13.19 ± 0.99 a	11.92 ± 0.80 b	14.30 ± 0.65 a
Ni (mg kg⁻¹)	0.95 ± 0.04 A	0.83 ± 0.03 B	0.94 ± 0.06 a	0.96 ± 0.04 a	0.84 ± 0.05 a	0.81 ± 0.03 a
Si (mg kg⁻¹)	591 ± 44.3 A	464 ± 24.4 B	542 ± 51.1 a	640 ± 71.9 a	430 ± 31.0 a	499 ± 36.0 a
Co (mg kg⁻¹)	0.156 ± 0.013 A	0.117 ± 0.010 B	0.137 ± 0.017 a	0.175 ± 0.019 a	0.110 ± 0.012 a	0.125 ± 0.016 a
Al (g kg⁻¹)	2.12 ± 0.17 A	1.59 ± 0.09 B	1.93 ± 0.19 a	2.30 ± 0.29 a	1.45 ± 0.11 a	1.73 ± 0.14 a

Values are means ± standard error. Different uppercase letters indicate significant differences between soil temperatures (24 °C *vs* 36 °C). Different lowercase letters indicate significant differences between water regimes (WW *vs* WD) within each temperature (Student's t-test, *$p < 0.05$).

Supplementary Figures:

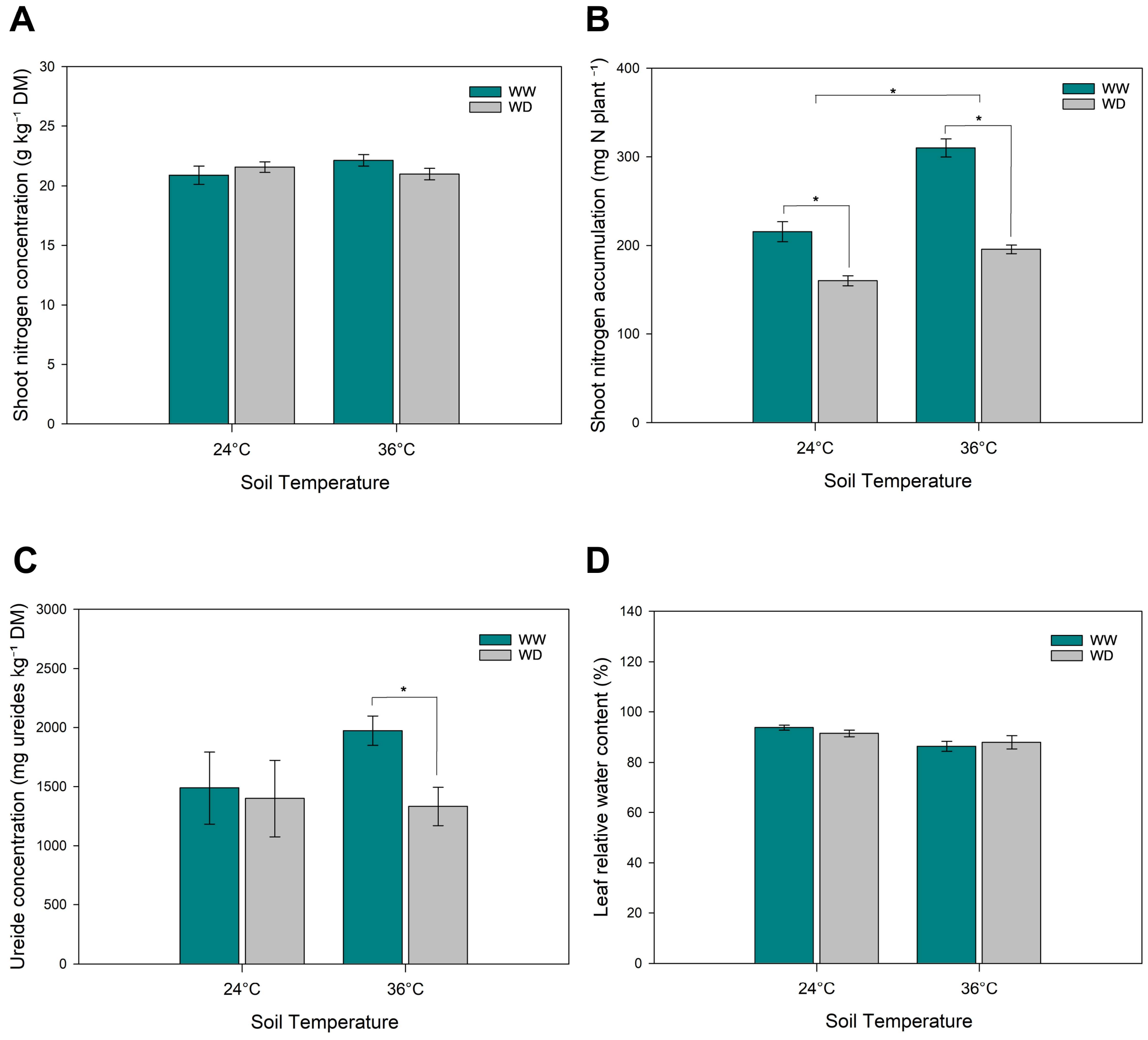

Suppl. Fig. S1 Effects of soil temperatures (24 °C and 36 °C) and water regimes (WW, well-watered; WD, water deficit) on shoot nitrogen status, ureide concentration, and leaf water relations. (A) Shoot nitrogen concentration (g kg^{-1} DM). (B) Shoot nitrogen accumulation (mg N $plant^{-1}$). (C) Ureide concentration in shoot dry matter (mg kg^{-1} DM). (D) Leaf relative water content (%). Bars represent mean ± standard error (n = 12). Asterisks indicate significant differences between treatments according to Student's *t* test ($p \leq 0.05$).

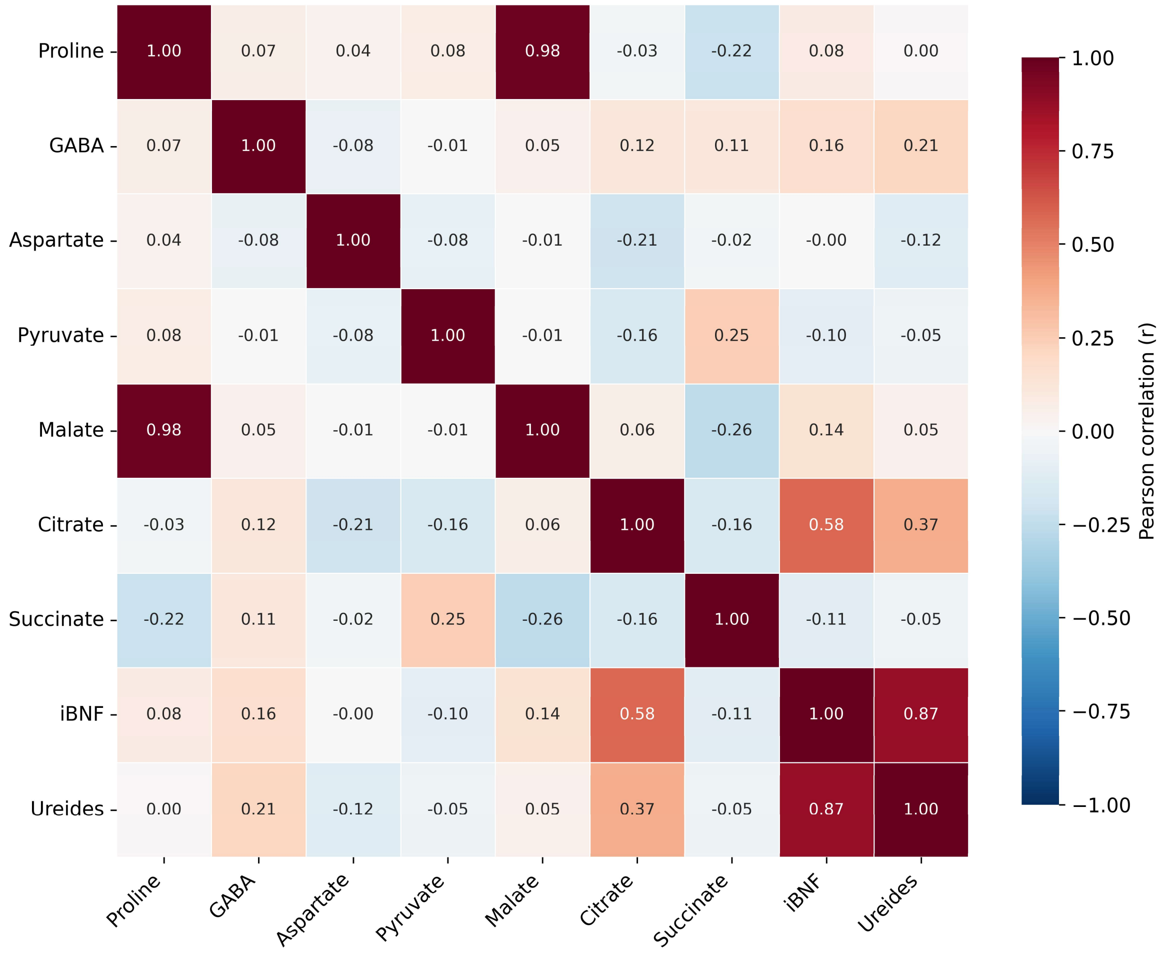

Suppl. Fig. S2 Integrated correlation matrix between metabolites and biological nitrogen fixation (BNF)-related variables in soybean plants. The color scale represents Pearson's correlation coefficient (r), ranging from −1 to +1, where positive values indicate direct correlations and negative values indicate inverse correlations.

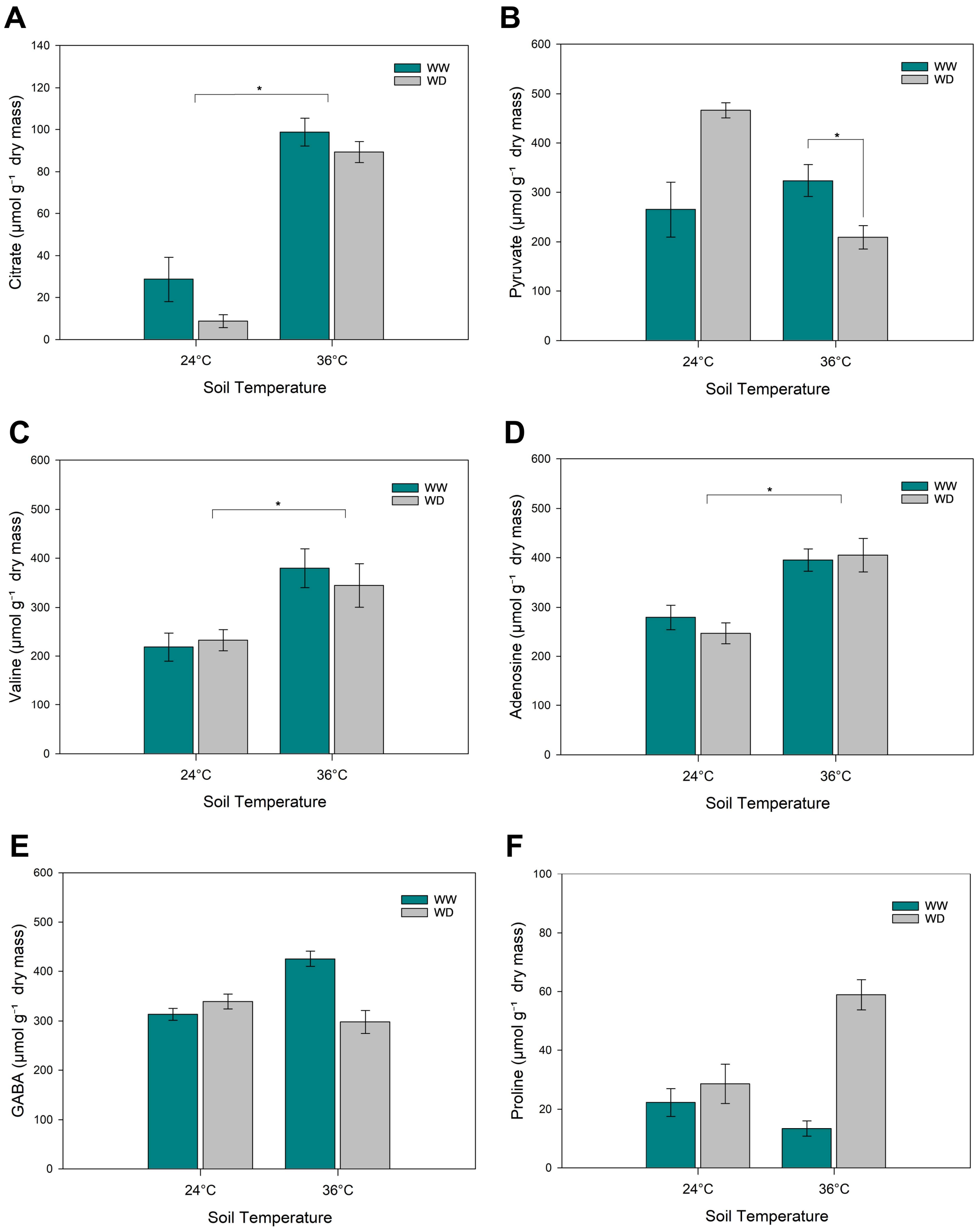

Suppl. Fig. S3 Effects of treatments on metabolite concentration in soybean. (A) Citrate. (B) Pyruvate. (C) Valine. (D) Adenosine. (E) GABA. (F) Proline. WW, well-watered; WD, water deficit. Values represent mean ± standard error (n = 12). Asterisks indicate significant differences between treatments according to Student's *t* test ($p \leq 0.05$).

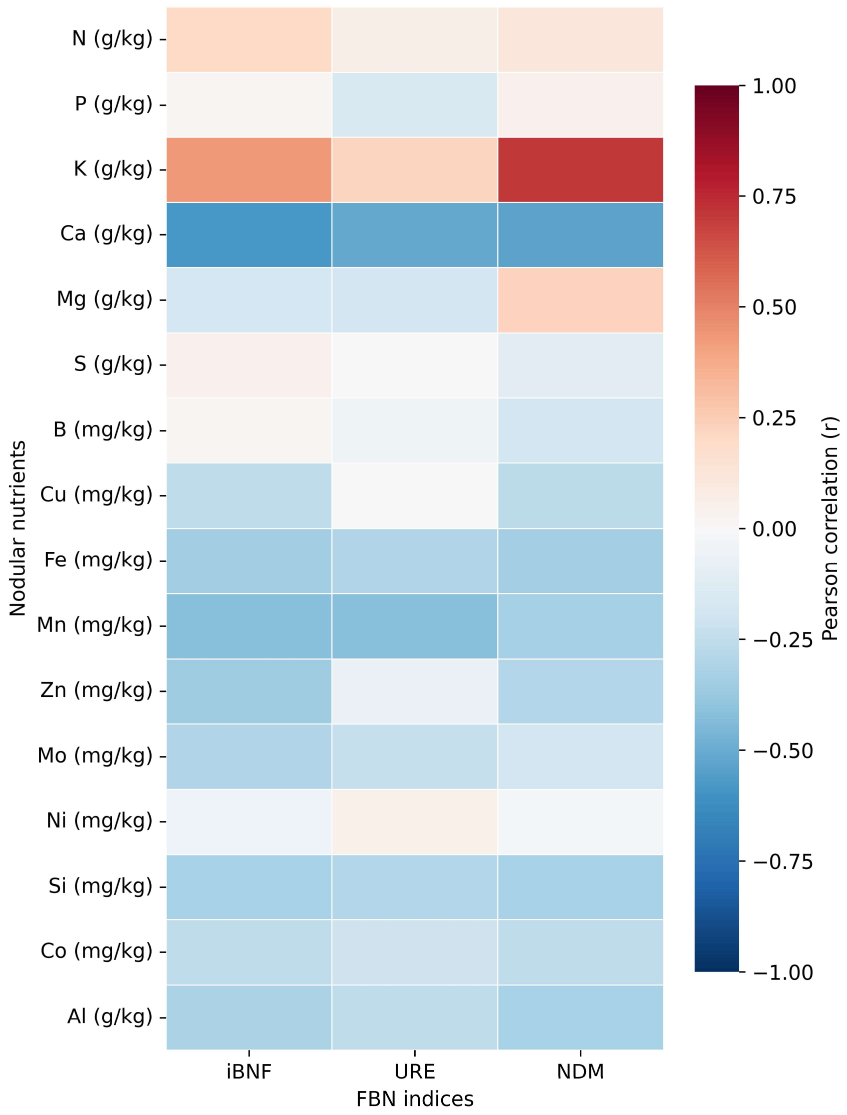

Suppl. Fig. S4 Correlation matrix between nodular nutrients and BNF indices in soybean. Correlations were calculated using Pearson's correlation coefficient (*r*). The color scale ranges from −1 (negative correlation) to +1 (positive correlation), indicating the strength and direction of associations. iBNF, integrated BNF efficiency index; URE, ureides; NDM, nodule dry mass.

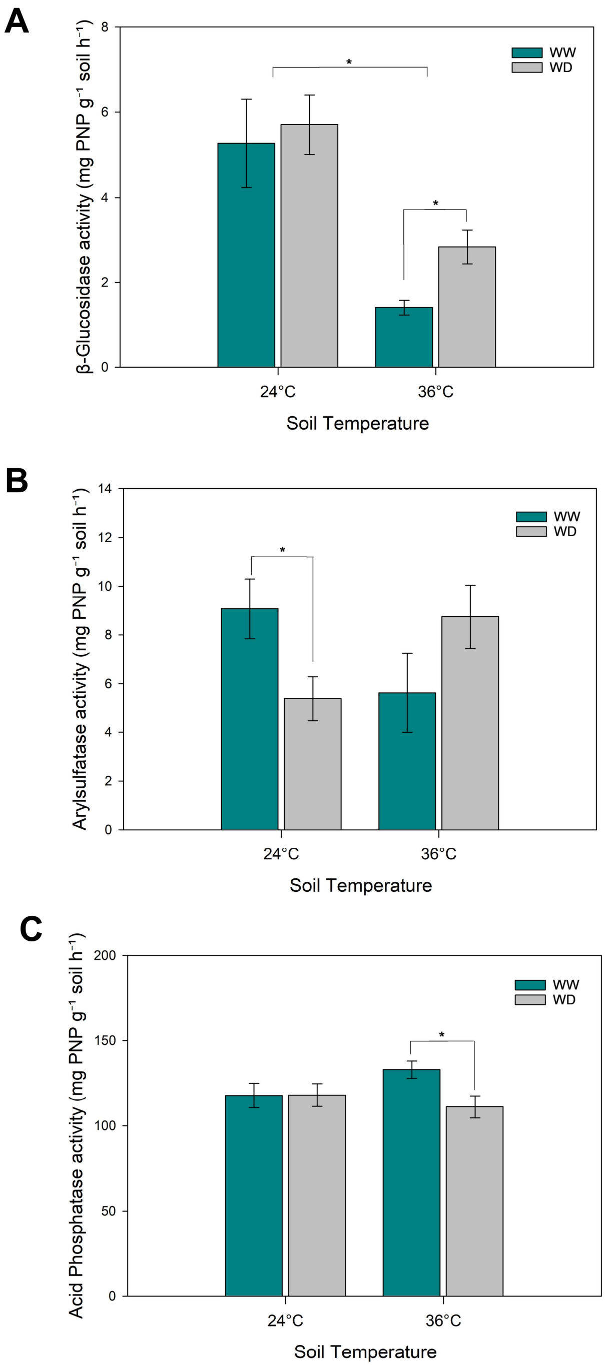

Suppl. Fig. S5 Effects of treatments on soil enzyme activities associated with nutrient cycling. (A) β-glucosidase. (B) Arylsulfatase. (C) Acid phosphatase. WW, well-watered; WD, water deficit. Values represent mean ± standard error (n = 12). Asterisks indicate significant differences between treatments according to Student's *t* test ($p \leq 0.05$).

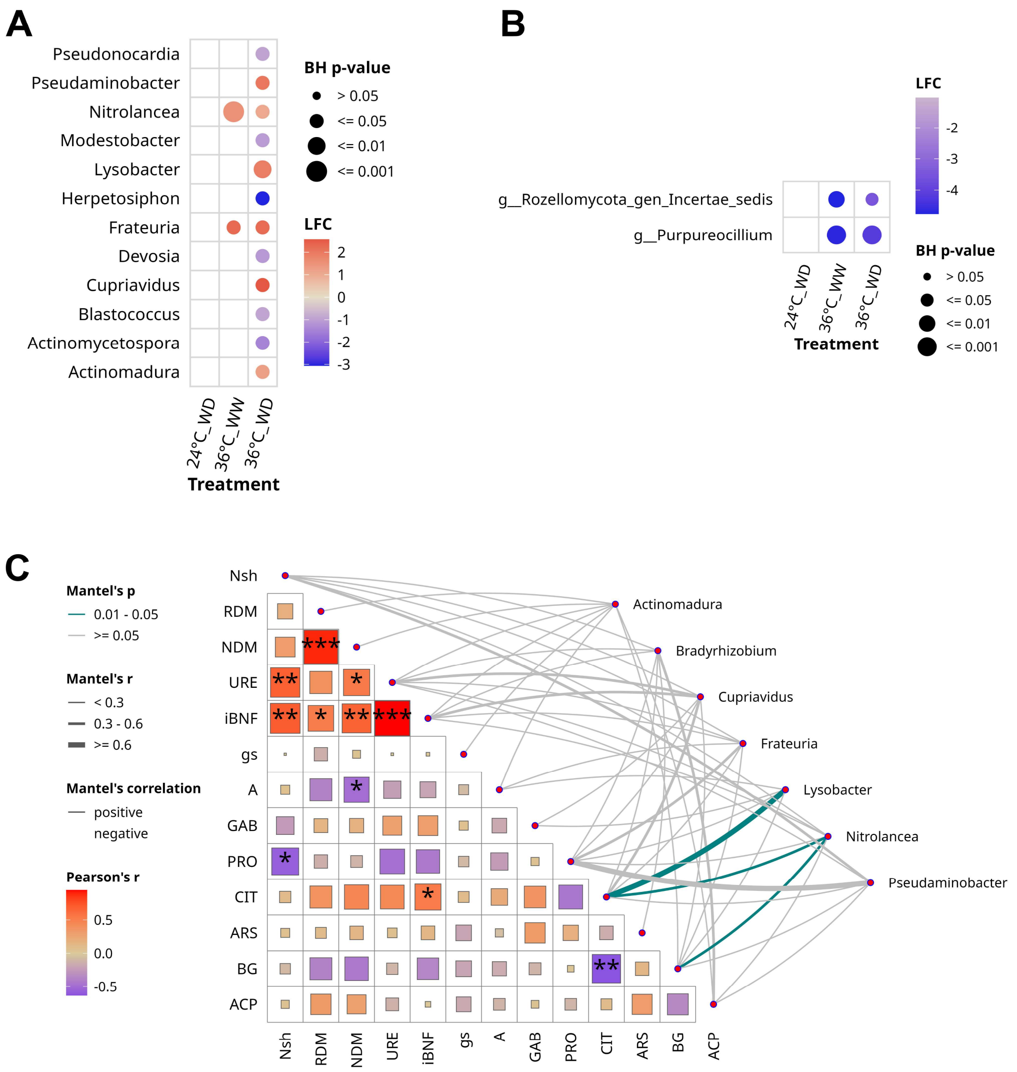

Suppl. Fig. S6 Differential abundance of bacterial (A) and fungal (B) taxa across treatments (24°C_WD, 36°C_WW, and 36°C_WD), using 24°C_WW as the control. Taxa responses are expressed as log fold change (LFC), indicating enrichment or depletion relative to the reference condition, with statistical significance determined by adjusted *p*-values. (C) Correlation network and matrix illustrating relationships among soil chemical attributes and dominant bacterial genera. Correlations are based on Pearson's coefficients, and significant associations are further supported by Mantel test results, where edge thickness reflects correlation strength and color denotes statistical significance.

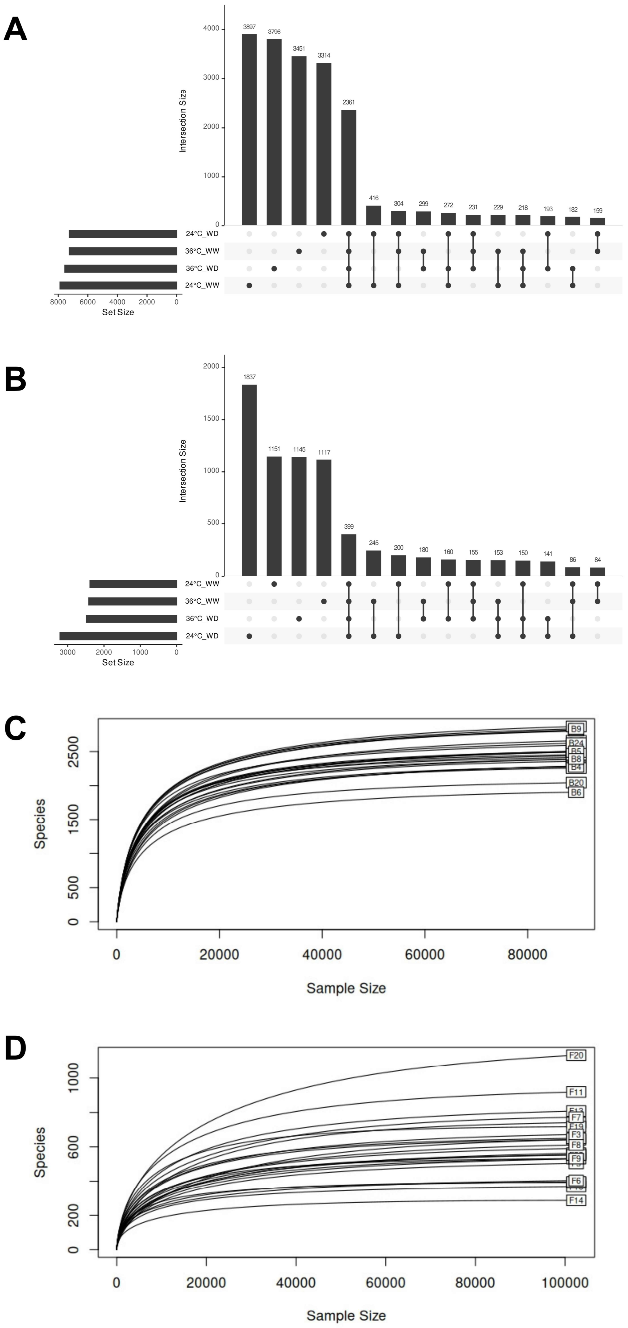

Suppl. Fig. S7 Analysis of microbial community richness and sampling depth. UpSet plots illustrating the number of shared and unique bacterial (A) and fungal (B) species across the different temperature and water treatments (24°C_WD, 36°C_WW, 36°C_WD, and the 24°C_WW control). Vertical bars represent the intersection size of species sets, while the matrix below indicates the specific treatment combinations compared. Rarefaction curves for bacterial (C) and

fungal (D) communities showing the number of observed species as a function of the number of sequences (sample size).
